# Testing the Linearity Assumption for Starch Structure-Property Relationships in Rices

**DOI:** 10.3389/fnut.2022.916751

**Published:** 2022-05-23

**Authors:** Yingting Zhao, Robert J. Henry, Robert G. Gilbert

**Affiliations:** ^1^Centre for Nutrition and Food Science, Queensland Alliance for Agriculture and Food Innovation, The University of Queensland, Brisbane, QLD, Australia; ^2^Jiangsu Key Laboratory of Crop Genetics and Physiology/State Key Laboratory of Hybrid Rice, College of Agriculture, Yangzhou University, Yangzhou, China; ^3^Jiangsu Key Laboratory of Crop Genomics and Molecular Breeding/Jiangsu Co-innovation Center for Modern Production Technology of Grain Crops, Yangzhou University, Yangzhou, China; ^4^Queensland Alliance for Agriculture and Food Innovation, The University of Queensland, Brisbane, QLD, Australia

**Keywords:** rice, linear correlation, starch, molecular structure, properties

## Abstract

Many properties of starch-containing foods are significantly statistically correlated with various structural parameters. The significance of a correlation is judged by the *p*-value, and this evaluation is based on the assumption of linear relationships between structural parameters and properties. We here examined the linearity assumption to see if it can be used to predict properties at conditions that are not close to those under which they were measured. For this we used both common domesticated rices (DRs) and Australian wild rices (AWRs), the latter having significantly different structural parameters and properties compared to DRs. The results showed that (1) the properties were controlled by more than just the amylopectin or amylose chain-length distributions or amylose content, other structural features also being important, (2) the linear model can predict the enthalpy ΔHg of both AWRs and DRs from the structural parameters to some extent but is often not accurate; it can predict the ΔHg of indica rices with acceptable accuracy from the chain length distribution and the amount of longer amylose chains (degree of polymerization > 500), and (3) the linear model can predict the stickiness of both AWRs and DRs to acceptable accuracy in terms of the amount of longer amylose chains. Thus, the commonly used linearity assumption for structure-property correlations needs to be regarded circumspectly if also used for quantitative prediction.

## Introduction

To understand and improve the processing and quality (including nutritional properties) of starch-containing foods, it is important to have statistically valid and physically meaningful correlations between starch structure and functional properties. These correlations are often obtained using the *p*-values of starch structure-property relations, with a correlation taken as significant if *p* is less than a chosen value, typically 0.05 or 0.01. This involves the assumption that the relation between the selected properties and structural parameters are linear. While mathematically this has to hold over sufficiently small changes in structure and properties (this is simply from Taylor's Theorem, which is universally valid), this assumption is likely to become invalid with large changes. It would be useful if one could use the coefficients resulting from these correlations predictively, e.g., to see what fraction of amylose (Am) long chains would produce a desired reduction in the rate of digestion of a starch-based food to glucose, as a guide to plant breeding and variety selection. A badly inaccurate prediction here could prove a costly error for a plant breeder.

Here we examine the predictive power of this common hypothesis for an important food system: the relation between structural parameters and functional properties (such as gelatinization behavior) for rice samples with diverse ranges of structures and properties.

Rice (*Oryza sativa* L.) is a widely consumed staple food and can be divided into two commonly domesticated rice species: *Oryza sativa* L. (from Asia), *Oryza glaberrima* Steud (from Africa), and 24 wild species (1). Clearly, when testing the predictive properties of parameters found using linear model, one wants to have as wide a range of structures as possible to improve the accuracy of these relationships. Compared to domesticated rices (DRs), Australian wild rices (AWRs) have a relatively broad genetic base, which results in significantly different starch molecular fine structure and properties in AWRs (2, 3), and together with DRs, provide a wide spectrum of structure and properties.

Starch, the major component of rice, accounts for 69–87% of the grain on a dry basis (4). Starch is a glucose polymer, mainly composed of two polymer variants: amylose (Am) and amylopectin (Ap). Am is largely linear with a few long-chain branches while Ap is highly branched. While starch has multiple levels of structure, most properties of interest are determined by the chain-length distribution (CLD) of starch (the fine structure) of both Ap and Am. To give one of many examples of properties significantly correlated with starch molecular fine structural parameters, the digestion rate of retrograded rice starch has been found to be mainly controlled by the distribution of short to medium Am chains (5). Earlier work (6–10) reported linear regression models and parameter values relating starch fine structure [or, in some cases, just amylose content (AC)] to gelatinization, retrogradation and texture properties for a range of rices. Although the relationships between rice properties and starch structures have been intensively investigated, it has been found (10–13) that the relationships between AC and eating quality are dependent on the ranges of AC variation, which suggests that the ranges of structure played an important role in determining properties. The rather different structural parameters between AWRs and domesticated rices therefore suggest that inclusion of both will be useful in a test of the predictability of the linearity assumption.

The aim of this study is to find linear correlations between structural parameters and certain properties and determine how well each property is predicted by a linear fit. This involves the following.

(1) The composition of three AWRs and 70 DRs were studied.
(2) The CLDs of rice starch (obtained after enzymatic debranching of the whole starch) were characterized with size-exclusion chromatography (SEC) and the results were fitted to biosynthesis-based models. These reduce the structural data to a small number of biosynthesis-related parameters suited to the types of analysis pursued here.
(3) We measured the thermal properties of starch, the *in-vitro* digestibility of cooked rice flours, which were subsequently fitted to kinetic models, and the texture of cooked rices. These treatments provide a small number of parameters describing these properties, which are suitable for the present aims.
(4) Linear regression was performed between properties and their related structural parameters.
(5)All parameters from the linear fits were then used to predict properties not included in the original data set, to test how well the linear regression treatment can be extrapolated beyond the conditions under which the linear coefficients were obtained.

## Materials and Methods

### Materials

Rice types used in this study consisted of AWRs (S01–S03) and DRs (S04–S73) (Supplementary Table 1). Varieties of DRs comprised *indica* (S04–S13, S24–S31, S40–S43, S46–S53, S61–S67) and *japonica* (S14–S23, S32–S39, S44–S45, S54–S60, S68–S73). For performing the linear regressions, here rice samples were grouped into three categories [Group A (three AWRs, 10 *indica* rices and 10 *japonica* rices), Group B (three AWRs and 10 *indica* rices), Group C (three AWRs and 10 *japonica* rices)] for each property. These are: for thermal properties, in Group A, sample AWR S01–S03, DR S04–S13, and S14–S23, in Group B, sample AWR S01–S03 and DR S04–S13, and in Group C, sample AWR S01–S03 and DR S14–S23; for *in vitro* digestion properties, in Group A, sample AWR S01–S03, DR S04, S06, S13, S28–S29, S31, S40–S43, and S14, S17, S19, S21, S23, S37–S39, S44–S45, in Group B, sample AWR S01–S03 and DR S04, S06, S13, S28–S29, S31, S40–S43, in Group C, sample AWR S01–S03 and DR S14, S17, S19, S21, S23, S37–S39, S44–S45; for textural properties, in Group A, sample AWR S01–S03, DR S13, S31, S40–S43, S46–S47, S52–S53, and S37–S38, S44, S54–S60, in Group B, sample AWR S01–S03 and DR S13, S31, S40–S43, S46–S47, S52–S53, in Group C, sample AWR S01–S03 and DR S37–S38, S44, S54–S60. After the linear regressions were performed, different samples were used in Groups A, B, and C (described below) to test the predictability of the linear regressions at conditions that were not close to those used to find the linear correlations. The order of DR varieties was determined by the order of their properties. AWR S01, *Oryza meridionalis* (Taxa B) (T.B.) was collected at Global Positioning System (GPS) latitude S 15°29'48.6” and GPS longitude E: 144°18'47.6”, in Queensland, Australia, on 11th May 2018. AWR S02, *Oryza officinalis* (O.O.) and AWR S03, *Oryza australiensis* (O.A.) were both provided by Australian GeneBank without detailed information. The AWR was dehulled manually, followed by polishing with a rice polisher (Model Kett, Tokyo, Japan). These three AWR varieties are the only ones currently available. Collecting sufficient amounts of samples for characterization is not straightforward, as they grow in the wild interspersed with many other plants, requiring laborious manual collection in a geographically remote location. In addition, the important need to request permission from traditional owners, following the legal protocols ensuring that their rights are protected, is time-consuming.

Some DR data [chain-length distributions (CLDs), and textural properties] were obtained from the literature from sources which used the same methodology, while the thermal properties, and *in-vitro* digestibility was measured as part of the present study. All sample details are shown in Supplementary Table 1.

The DR starches, obtained under the same conditions as the AWR starches, were as follows. From Li et al. (14): *indica* rice variety: S13, S31, S40–S43, S46–47, S52–53, S61–62; *japonica* rice variety: S37–S38, S44, S54–S60, S68–S72. From Cheng Li and co-workers (unpublished; University of Shanghai for Science and Technology): *indica* rice varieties S63–S67. From Li et al. (unpublished, Yangzhou University): *japonica* rice varieties S39, S45, S73. From Zhu et al. (*indica* rice varieties S48–S51) (15). The collection details of all DRs are summarized in Supplementary Table 1. DR S04–S09, S11–S12, S14–S30, and S32–S36 were provided by the Suzhou Seed Store Center, China, in 2019, DR S10, S48–S51, S63–S67 were planted and harvested in PoLiu village, Lingshui County, Hainan Province, China in 2017, and DR S13, S31, S37–S47, S52–S62, S68–S73 were planted and harvested at the experimental field in Yangzhou University, Yangzhou City, Jiangsu Province, China in 2018. The DR kernels were dehusked using a rice huller (Model SY88-TH, Korea) and then polished with a rice polisher. Both AWRs and DRs were stored at 4°C in air-tight plastic bags before use.

Protease (from *Streptomyces griseus* type XIV) (P5147, ≥ 3.5 Units/mg solid), α-amylase from human saliva (A1031, 84 units/mg solid), pepsin from porcine gastric mucosa (P6887, 3,200–4,500 Units/mg protein), and pancreatin from porcine pancreas (P7545, 8 × USP) were purchased from Sigma-Aldrich Chemical Co. (St. Louis, MO, USA). Amyloglucosidase (from *Aspergillus niger*) (3,260 Units/mL) and isoamylase (from *Pseudomonas* sp.) (200 Units/mL) were purchased from Megazyme International Ltd. (Bray, Co. Wicklow, Ireland). Pullulan SEC standards with known peak molecular weights ranging from 180 to 1.22 × 10^6^ were obtained from Polymer Standards Service (PSS) GmbH (Mainz, Germany). Other chemical reagents were analytical grade and used as received.

### Composition of Rice Grains

The total starch content was measured as previously described using a Megazyme total starch (AA/AMG) assay kit (16). The crude protein content was measured using a Leco CNS-2000 analyzer (Seminole, Florida, USA) (by the combustion method), and then calculated from the nitrogen content with a conversion factor of 5.95.

### Starch Extraction From Rice Grains

The extraction of starch from the rice grains followed a previously described method (16). Briefly, rices were ground into flour using a cryo-grinder (MM400, Netsch, Germany, 10 s at a time, 6 times at 20 s^−1^) before being filtered with a 75-μm sieve. The flour was immersed in 0.45% sodium metabisulfite solution (the volume ratio of rice flour to solution being 1:3) at 4°C for 0.5 h. Proteins were removed by protease treatment [2.5 Units mL^−1^ of protease in tricine buffer (250 mM, pH 7.5)] at 37°C overnight. After centrifuging at 4,000 × *g* for 10 min, the supernatant was discarded. The treated flour was washed with deionized water six times, then twice precipitated in ethanol. Finally, the extracted starch was freeze-dried (SP Scientific, VirTis, BTP-9ESOOX, U.K.) for 48 h.

### Characterization of Starch Molecular Structure

SEC was used to measure the CLDs of debranched starches as described in detail previously (17). SEC separates polymer molecules by molecular size, specifically the hydrodynamic radius (*R*_h_). Briefly, native starch was dissolved in dimethyl sulfoxide (DMSO) with 0.5% (w/w) LiBr (DMSO/LiBr) at 80°C before centrifugation, the supernatant then mixed with absolute ethanol, and the resulting precipitate was debranched with isoamylase prior to freeze-drying. The freeze-dried sample was dissolved in DMSO/LiBr and the resulting supernatant was then injected into the SEC. The CLDs were obtained using a LC20AD SEC system (Shimadzu Corporation, Kyoto, Japan) equipped with three columns in sequence (PSS, Mainz, Germany): GRAM pre-column, GRAM 100 and GRAM 1000 columns, and a RID-10A refractive index detector (Shimadzu Corporation, Kyoto, Japan). DMSO/LiBr solution was used as the mobile phase with a flow rate of 0.6 mL/min.

### Fitting Ap and Am CLDs to Models

The Ap and Am starch CLDs were each fitted to biosynthesis-based models using publicly available codes. This enables a reduction of the CLD data into a small number of biologically-relevant parameters, suitable for finding statistically valid correlations (18, 19). Briefly, both models divide the Ap and Am components in the CLD into different regions with different DP ranges (in the present case, three regions for Ap and two or three regions for Am, depending on CLD features such as peaks and shoulders). The chains in each region are assumed to be predominantly but not exclusively synthesized by a given “enzyme set.” Each enzyme set contains an isoform of starch synthase (SS), one or two isoforms of starch branching enzyme (SBE) and an isoform of starch debranching enzyme (DBE). For enzyme set *i*, the contribution to the CLD from this set can be calculated from the values of two parameters: β_Am, i_ and *h*_Am, i_ (*i* = 1, 2, …) for Am and β_Ap, i_ and *h*_Ap, i_ (*i* = 1, 2, 3) for Ap. The β_*i*_ are the ratios of the activity of SBE to that of SS in set *i* and the *h*_*i*_ are the relative activities of the SS in the enzyme set.

### Thermal Properties

The gelatinization properties of rice starches were measured by differential scanning calorimetry (DSC; Netzsch, Ahlden, Germany). Starch (5 mg, dry weight basis) and water (at a weight ratio of 1:2) were weighed in a DSC aluminum crucible and sealed. After equilibrating at 4°C for 24 h and then at room temperature for 1 h, the crucibles were held at 20°C for 1 min in the DSC oven, and then heated from 20 to 100°C at a rate of 10°C/min, followed by cooling at 20°C/min from 100 to 20°C. An empty crucible was used as a reference. The onset temperature (*T*_o_), peak temperature (*T*_p_), conclusion temperature (*T*_c_) and crystal melting enthalpy (Δ*H*_g_) were measured.

### *In vitro* Digestion

An *in-vitro* digestion method used, namely that of Zhao et al. (3). Briefly, rice flour [0.5–1.27 mm, mimicking size during mastication (3), ~70 mg] was cooked with 2 mL of distilled water in 50-mL centrifuge tube in a boiling water bath for 30 min and then incubated in a 37°C water bath. Then 0.2 mL artificial saliva solution (250 U/mL pancreatic α-amylase in carbonate buffer at pH 7 containing 21.1 mM KCl, 1.59 mM CaCl_2_, and 0.2 mM MgCl_2_) was added to each tube and incubated for 20 s, followed by incubation with porcine pepsin (1 mg/mL) in HCl solution (1 mL, 0.02 M) for 30 min. The digesta were then neutralized with 1 mL NaOH (0.02 M) and mixed with 5 mL sodium acetate buffer (pH 6, 0.2 M) containing 200 mM CaCl_2_, 0.49 mM MgCl_2_ and 0.02% w/v NaN_3_. Pancreatin (2 mg/mL) and 28 U/mL amyloglucosidase in the same sodium acetate buffer solution (1 mL) were added to the digesta. After times ranging from 0 to 300 min, a 0.1 mL aliquot of solution was quickly transferred to a microcentrifuge tube containing 0.9 mL absolute ethanol, to stop the reaction. The glucose content of each sample was determined by the GOPOD method with a Megazyme D-Glucose (County Wicklow, Ireland) assay kit. The whole digestion progress was carried out at 37°C with constant shaking at 100 rpm. The *in vitro* digestion results were first fitted to a first order equation:


(1) $$\begin{array}{r} C\left(t\right)\;=\;C\infty \;\left(1-{\text{e}}^{kt}\right) \end{array}$$

Here *C*(*t*) is the fraction of total starch digested at time *t*, *C*_∞_ is the fraction of starch digested at very long reaction time and *k* is the digestion rate coefficient of the starch. The digestion curves were further treated with the logarithm-of-slope (LoS) method, which identifies the region(s) following first-order loss kinetics (20), followed by being treated with two other methods [the non-linear least-squares (NLLS) method (21), and a parallel digestion model (22)] which are both based on the same principles as the LoS method and yield more precise values of the parameters *k* and *C*_∞_.

### Textural Properties

The textural properties of cooked rice grains were measured using a texture analyzer (TA.XT plus, Stable Micro Systems Ltd., London, UK), equipped with a P36R cylindrical probe, following a previously described method (14). Briefly, polished AWR grains were cooked with MilliQ water (rice: water weight ratio 1:1.6) in a rice cooker. After cooling to room temperature, a 1 g cooked AWR sample was placed on the sample plate of the texture analyzer with single-grain thickness. A compression force program up to 70 % strain was applied to lower and withdraw the probe, at a speed of 1 mm/s to mimic oral chewing. This will of course break some or all of the rice cooked grains, as happens during chewing. Texture measurements were performed five times on the cooked AWR grains at room temperature.

The textural data of cooked DRs, obtained under the same conditions as the cooked AWRs, were those provided by the same sources as those providing the samples, detailed above.

### Linear Regression

Three groups of starches {Group A [three AWRs and 20 domesticated rices (DRs)], Group B (three AWRs and 10 *indica* rices), Group C (three AWRs and 10 *japonica* rices)} (mentioned above) were used to build linear correlations between properties parameters and their related structural parameters by a backward multiple linear regression (related structural parameters of each property are from the literature) (4, 5, 14, 23–34). Two mathematical biosynthesis-based models for obtaining parameters were used to parameterize the data. Since the starch granular and crystalline structures are greatly disrupted by the cooking process, only grain composition and starch molecular structures need be considered here. The linearity assumption assumes that the *i*^th^ property *P*_*i*_ is given in terms of structural parameters *s*_*j*_ by:


(2) $$\begin{array}{r} P_{i}\;=\;\underset{j}{\sum }\;a_{ij}s_{j} \end{array}$$

Here the *a*_*ij*_ are constants whose values depend on the values of the *s*_*j*_ and *P*_*i*_. Non-linear behavior would be when one has to include terms in *s*_*i*_ *s*_*j*_, $s_{j}^{2}$ etc. to fit or to predict data with desired accuracy. Using very different types of starch (domesticated and wild varieties, in the present case) is useful for testing the extrapolation of linear parameterization to larger differences: different types of starch will have a wide “space” of structures and thereby a wider data range for improved testing of this assumption. After performing these linear regressions (*p* < 0.05) on the subset of our samples given above, another 16 DRs (8 *indica* and 8 *japonica*), were treated in the same way as the samples used to build the linear regressions and used in Groups A, B, and C, respectively. For thermal properties, sample DR S24–S31, and S32–S39 were used in Group A, samples DR S24–S31 were used in Group B, and samples DR S32–S39 were used in Group C. For *in vitro* digestion properties, samples DR S05, S10, S46–S51, and S18, S20, S22, S32–S36 were used in Group A, samples DR S05, S10, S46–S51 were used in Group B, and samples DR S18, S20, S22, S32–S36 were used in Group C. For textural properties, samples DR S10, S61–S67, and S39, S45, S68–S73 were used in Group A, samples DR S10, S61–S67 were used in Group B, and samples DR S39, S45, S68–S73 were used in Group C. These last were used to test the predictability of the linear regressions over significant displacements from the conditions used to find the linear correlations.

It is noted that this paper concerns structure-property relations; how these structures were formed (different growth conditions, etc.) is irrelevant to the present aims, although of interest in other contexts.

### Statistical Analysis

Statistical analysis was conducted using SPSS software (version 28.0, SPSS Inc., Chicago, IL, USA). Analysis of Variance (ANOVA) was used to determine significant differences in starch molecular fine structural parameters, moisture content, AC and property parameters. A backward regression approach was employed in the multiple linear regression, and coefficient of determination (*R*^2^) and root mean square errors (RMSE) were used as indicator of significance for the regression models.

## Results and Discussion

### Compositions of Rice Grains

The chemical compositions of both AWRs and DRs are presented in Supplementary Table 1. No significant differences were seen in total starch content, total crude protein content and AC of AWRs compared to those of the DR samples. Since starch and protein together accounted for up to 92.5% on a dry basis, lipid content was not considered in linear models in this paper, but could be considered in future work to see if predictability could be improved.

### Comparison of Starch Molecular Structural Parameters of AWRs and DRs

**Figure 1:**
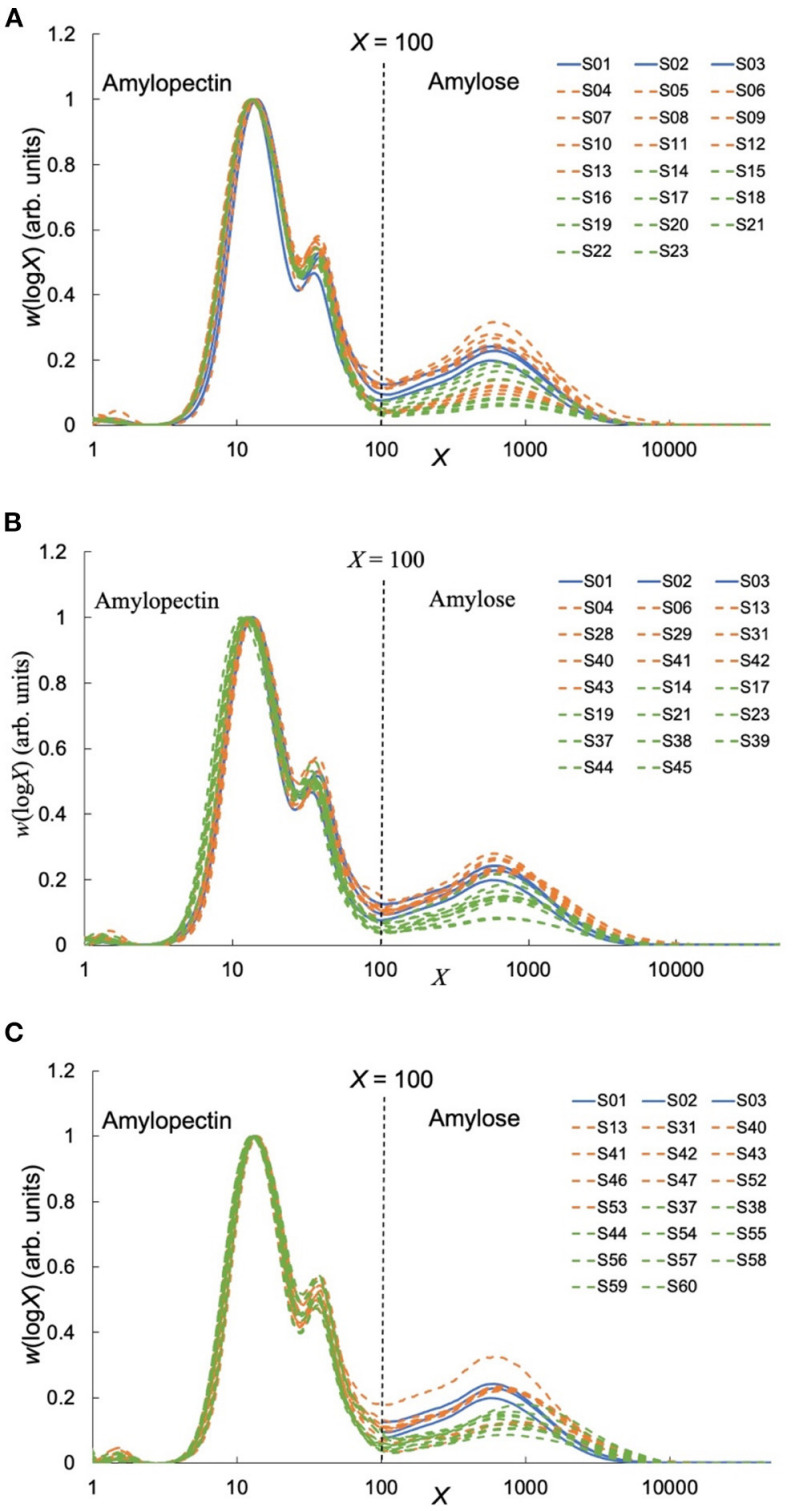
Size-Exclusion Chromatography (SEC) results. Values of *w*(log*X*), of debranched starch from AWRs and DRs. **(A)** Results used to build structure-thermal properties relations, **(B)** results used to build structure-*in vitro* digestion properties relations, **(C)** results used to build structure-textural properties relations. The debranched DR starch data obtained under the same conditions as the AWR starches are retrieved from the literature (Supplementary Table 1). AWRs consist of S01–S03, *indica* variety contains S04–S13, S28–S29, S31, S40–S43, S46–S47, S52–S53 and *japonica* variety contains S14–S23, S37–S39, S44–S45, S54–S60, respectively.

SEC weight distributions of debranched rice starch are shown in Figure 1, plotted as the weight distributions *w*(log*X*), which are the weight (not molecular weight) of chains as a function of their degree of polymerization (DP) *X*. Normalization of such distributions is arbitrary; here, the data were normalized to the highest Ap branch peak. Generally, chains shorter than DP 100 are taken as Ap branches and those longer than DP 100 as Am branches (18). All rice varieties have similar CLDs: two large Ap peaks and one small Am peak, as commonly seen. The first Ap peak around DP 13–14 are Ap chains confined to a single lamella in the native starch, while the second Ap peak about DP 36–38 are trans-lamellae Ap chains traversing two or more lamella in the native starch. These Ap and Am CLDs were parameterized using two biosynthesis-based models (18, 19). Fitting results for AWRs and typical examples of one *indica* rice and one *japonica* rice are presented in Supplementary Figure 1, and all model-based fitting parameters used to build structure-property relations are given in Tables 1–3. Biosynthesis of shorter Ap chains is dominated by the model applied to “Ap region 1,” with the model parameters β_Ap, 1_ and *h*_Ap, 1_, medium ones by β_Ap, 2_ and *h*_Ap, 2_, and longer ones by β_Ap, 3_ and *h*_Ap, 3_. Biosynthesis of shorter Am chains are dominated by “Am region 1” with parameters β_Am, 1_ and *h*_Am, 1_, and longer ones by β_Am, 2_ and *h*_Am, 2_. Large differences among the CLD fitting parameters were observed for different starches.

(1) In the SEC results used to build structure-thermal properties relations (Figure 1A and Table 1), generally, AWR starches had higher β_Am, 2_ than those of domesticated ones, consistent with AWR starches having shorter Am long chains (especially fewer *X* > 5,000 in AWR). This lower amount for *X* > 5,000 in AWR starches could be due to the lower enzyme activity of an isoform of granule-bound starch synthase (19).
(2) The SEC results (Figure 1B and Table 2) showed that AWR starches used here also had higher β_Am, 2_ than those of domesticated ones. The *h*_Am, 1_ and *h*_Am, 2_ values were generally in the order *indica* rice starches>AWR starches>*japonica* rice starches, which might be because of the different activity of an isoform of SS in different starches (19).
(3) The SEC results used to build structure-textural properties relations (Figure 1C and Table 3) showed that, generally, AWR starches had both higher β_Am, 1_ and higher β_Am, 2_ compared to domesticated ones.

**Table 1:** Structural parameters of rice starches for building linear regression models of structure-thermal properties.

**Samples**	**Amylopectin fitting parameters**						**Amylose fitting parameters**			
	**β_Ap, 1_/0.01**	**β_Ap, 2_/0.01**	**β_Ap, 3_/0.01**	***h*_Ap, 1_/0.01**	***h*_Ap, 2_/0.01**	***h*_Ap, 3_/0.01**	**β_Am, 1_/0.001**	**β_Am, 2_/0.001**	***h*_Am, 1_/0.001**	***h*_Am, 2_/0.001**
S01	13.3 ± 0.0^bc^	6.9 ± 0.0^fg^	2.7 ± 0.0^ghi^	94.4 ± 0.0^a^	3.6 ± 0.0^a^	0.17 ± 0.00^de^	26.7 ± 0.2^c^	3.0 ± 0.1^ab^	47.1 ± 0.8^e^	177.6 ± 1.0^ef^
S02	13.2 ± 0.0^bcd^	6.6 ± 0.0^i^	2.3 ± 0.0^j^	93.3 ± 0.2^a^	3.6 ± 0.0^a^	0.21 ± 0.00^a^	26.0 ± 0.4^cde^	3.0 ± 0.0^ab^	73.7 ± 0.2^a^	187.4 ± 2.7^de^
S03	13.6 ± 0.0^a^	7.1 ± 0.1^e^	2.9 ± 0.2^fgh^	80.1 ± 0.8^de^	2.9 ± 0.0^g^	0.13 ± 0.00^f^	24.7 ± 2.9^cdefgh^	3.1 ± 0.0^a^	37.2 ± 0.1^f^	153.6 ± 4.3^g^
S04	13.3 ± 0.6^bc^	6.8 ± 0.1^gh^	2.5 ± 0.0^ij^	81.1 ± 3.1^d^	3.4 ± 0.2^b^	0.16 ± 0.01^e^	25.5 ± 0.2^cdefg^	2.9 ± 0.0^bc^	55.6 ± 2.3^bc^	218.2 ± 3.0^b^
S05	12.7 ± 0.0^gh^	6.7 ± 0.0^hi^	2.6 ± 0.0^hij^	79.6 ± 0.1^e^	3.3 ± 0.0^c^	0.17 ± 0.00^de^	25.9 ± 1.9^cdef^	2.8 ± 0.0^cd^	58.4 ± 7.1^b^	207.3 ± 10.0^c^
S06	12.6 ± 0.0^h^	6.6 ± 0.0^i^	2.5 ± 0.0^ij^	76.9 ± 0.0^fgh^	3.2 ± 0.0^d^	0.18 ± 0.00^cd^	26.9 ± 0.8^bc^	2.9 ± 0.1^bc^	69.6 ± 4.9^a^	245.8 ± 2.8^a^
S07	12.9 ± 0.0^efg^	7.3 ± 0.0^d^	3.5 ± 0.1^abc^	77.5 ± 0.1^fg^	3.1 ± 0.0^e^	0.10 ± 0.00^gh^	23.4 ± 1.9^defghi^	2.7 ± 0.1^de^	17.4 ± 0.3^ij^	91.5 ± 6.9^kl^
S08	12.9 ± 0.0^efg^	7.4 ± 0.0^cd^	3.5 ± 0.1^abc^	78.1 ± 0.1^f^	3.1 ± 0.0^e^	0.10 ± 0.00^gh^	24.9 ± 1.3^cdefgh^	2.5 ± 0.0^fg^	18.6 ± 1.9^ij^	108.1 ± 8.7^j^
S09	13.0 ± 0.0^def^	7.4 ± 0.0^cd^	3.5 ± 0.0^abc^	76.4 ± 0.2^ghi^	3.0 ± 0.0^f^	0.10 ± 0.00^gh^	26.1 ± 1.7^cd^	2.5 ± 0.1^fg^	17.3 ± 1.3^ij^	84.0 ± 7.2^lm^
S10	13.4 ± 0.0^ab^	6.8 ± 0.2^gh^	3.3 ± 0.7^cde^	86.3 ± 0.1^c^	3.4 ± 0.0^b^	0.20 ± 0.03^ab^	22.1 ± 1.3^hijk^	3.0 ± 0.0^ab^	51.9 ± 6.4^cd^	193.5 ± 13.0^d^
S11	13.0 ± 0.0^def^	7.4 ± 0.0^cd^	3.7 ± 0.1^ab^	76.9 ± 0.2^fgh^	3.0 ± 0.0^f^	0.10 ± 0.00^gh^	33.1 ± 4.1^a^	2.6 ± 0.1^ef^	23.8 ± 0.6^h^	95.3 ± 4.7^k^
S12	12.8 ± 0.0^fgh^	7.3 ± 0.0^d^	3.8 ± 0.1^a^	78.0 ± 0.4^f^	3.2 ± 0.0^d^	0.11 ± 0.00^g^	25.5 ± 0.4^cdefg^	2.5 ± 0.1^fg^	17.5 ± 0.1^ij^	75.1 ± 5.1^mn^
S13	12.9 ± 0.0^efg^	6.9 ± 0.0^fg^	2.5 ± 0.0^ij^	90.1 ± 1.9^b^	3.4 ± 0.0^b^	0.19 ± 0.00^bc^	20.2 ± 0.5^jk^	2.5 ± 0.0^fg^	48.3 ± 0.1^de^	173.6 ± 0.0^f^
S14	13.2 ± 0.0^bcd^	7.6 ± 0.1^ab^	3.6 ± 0.0^abc^	75.8 ± 0.3^hij^	2.8 ± 0.0^h^	0.09 ± 0.00^hi^	19.3 ± 1.1^k^	2.5 ± 0.1^fg^	15.7 ± 0.5^ij^	62.9 ± 0.0°
S15	13.2 ± 0.0^bcd^	7.6 ± 0.0^ab^	3.8 ± 0.1^a^	75.0 ± 0.1^ij^	2.7 ± 0.0^i^	0.07 ± 0.00^j^	23.0 ± 2.8^efghij^	2.4 ± 0.2^g^	13.6 ± 0.9^j^	47.9 ± 0.0^p^
S16	13.2 ± 0.0^bcd^	7.7 ± 0.0^a^	3.7 ± 0.1^ab^	76.2 ± 0.1^ghij^	2.7 ± 0.0^i^	0.08 ± 0.00^ij^	21.3 ± 1.8^ijk^	2.5 ± 0.1^fg^	14.2 ± 0.7^ij^	52.3 ± 1.9^p^
S17	13.3 ± 0.0^bc^	7.5 ± 0.0^bc^	3.3 ± 0.0^cde^	76.5 ± 0.2^ghi^	2.9 ± 0.0^g^	0.10 ± 0.00^gh^	29.6 ± 1.2^b^	2.6 ± 0.1^ef^	25.7 ± 2.0^h^	129.0 ± 15.9^i^
S18	13.0 ± 0.0^def^	7.0 ± 0.0^ef^	3.0 ± 0.1^efg^	76.0 ± 0.3^ghij^	3.1 ± 0.0^e^	0.13 ± 0.00^f^	22.9 ± 0.4^fghij^	2.8 ± 0.1^cd^	30.9 ± 2.5^g^	150.7 ± 5.1^gh^
S19	13.1 ± 0.0^cde^	7.6 ± 0.0^ab^	3.4 ± 0.0^bcd^	76.1 ± 0.4^ghij^	2.9 ± 0.0^g^	0.10 ± 0.00^gh^	27.4 ± 1.7^bc^	2.7 ± 0.0^de^	19.2 ± 2.7^i^	110.0 ± 1.2^j^
S20	13.2 ± 0.0^bcd^	7.0 ± 0.1^e^	3.1 ± 0.1^def^	76.4 ± 0.0^ghi^	3.1 ± 0.0^e^	0.11 ± 0.02^g^	22.7 ± 0.4^ghij^	2.7 ± 0.1^de^	27.2 ± 3.8^gh^	141.5 ± 8.3^h^
S21	13.2 ± 0.0^bcd^	7.5 ± 0.0^bc^	3.6 ± 0.1^abc^	74.8 ± 0.2^j^	2.8 ± 0.0^h^	0.08 ± 0.00^ij^	20.1 ± 1.1^jk^	2.5 ± 0.1^fg^	16.0 ± 0.1^ij^	63.7 ± 4.7°
S22	13.3 ± 0.0^bc^	7.7 ± 0.0^a^	3.8 ± 0.2^a^	76.1 ± 0.3^ghij^	2.8 ± 0.0^h^	0.08 ± 0.00^ij^	21.3 ± 0.2^ijk^	2.4 ± 0.0^g^	17.0 ± 0.8^ij^	64.5 ± 1.5^no^
S23	13.3 ± 0.0^bc^	7.7 ± 0.2^a^	3.6 ± 0.2^abc^	75.2 ± 0.2^ij^	2.7 ± 0.0^i^	0.08 ± 0.00^ij^	19.4 ± 0.7^k^	2.6 ± 0.2^ef^	15.8 ± 0.9^ij^	66.0 ± 3.9^no^

*Mean ± standard deviation is calculated from duplicate measurements; values with different letters in the same column are significantly different at p < 0.05*.

**Table 2:** Structural parameters of rice starches for building linear regression models of structure-*in vitro* digestion properties.

**Samples**	**Amylopectin fitting parameters**						**Amylose fitting parameters**			
	**β_Ap, 1_/0.01**	**β_Ap, 2_/0.01**	**β_Ap, 3_/0.01**	***h*_Ap, 1_/0.01**	***h*_Ap, 2_/0.01**	***h*_Ap, 3_/0.01**	**β_Am, 1_/0.001**	**β_Am, 2_/0.001**	***h*_Am, 1_/0.001**	***h*_Am, 2_/0.001**
S01	13.3 ± 0.0^efgh^	6.9 ± 0.0^fg^	2.7 ± 0.0^def^	94.4 ± 0.0^abc^	3.6 ± 0.0^bc^	0.17 ± 0.00^fg^	26.7 ± 0.2^cd^	3.0 ± 0.1^ab^	47.1 ± 0.8^e^	177.6 ± 1.0^ef^
S02	13.2 ± 0.0^fgh^	6.6 ± 0.0^h^	2.3 ± 0.0^fg^	93.3 ± 0.2^bc^	3.6 ± 0.0^bc^	0.21 ± 0.00^bc^	26.0 ± 0.4^cde^	3.0 ± 0.0^ab^	73.7 ± 0.2^b^	187.4 ± 2.7^d^
S03	13.6 ± 0.0^bcd^	7.1 ± 0.1^e^	2.9 ± 0.2^cd^	80.1 ± 0.8^def^	2.9 ± 0.0^gh^	0.13 ± 0.00^j^	24.7 ± 2.9^ef^	3.1 ± 0.0^a^	37.2 ± 0.1^g^	153.6 ± 4.3^g^
S04	13.3 ± 0.6^efg^	6.8 ± 0.1^g^	2.5 ± 0.0^defg^	81.1 ± 3.1^de^	3.4 ± 0.2^cde^	0.16 ± 0.01^gh^	25.5 ± 0.2^de^	2.9 ± 0.0^bc^	55.6 ± 2.3^d^	218.2 ± 3.0^b^
S06	12.6 ± 0.0^j^	6.6 ± 0.0^h^	2.5 ± 0.0^defg^	76.9 ± 0.0^efg^	3.2 ± 0.0^ef^	0.18 ± 0.00^ef^	26.9 ± 0.8^cd^	2.9 ± 0.1^bc^	69.6 ± 4.9^c^	245.8 ± 2.8^a^
S13	12.9 ± 0.0^i^	6.9 ± 0.0^fg^	2.5 ± 0.0^defg^	90.1 ± 1.9^c^	3.4 ± 0.0^cde^	0.19 ± 0.00^de^	20.2 ± 0.5^i^	2.5 ± 0.0^fg^	48.3 ± 0.1^e^	173.6 ± 0.0^f^
S28	14.3 ± 0.0^a^	7.0 ± 0.0^ef^	2.7 ± 0.0^def^	93.0 ± 0.4^bc^	3.7 ± 0.0^b^	0.15 ± 0.00^hi^	23.0 ± 1.2^fg^	2.8 ± 0.0^cd^	40.2 ± 0.4^f^	205.2 ± 8.4^c^
S29	13.6 ± 0.2^bcd^	7.0 ± 0.1^ef^	2.6 ± 0.0^defg^	97.9 ± 2.4^ab^	3.7 ± 0.1^b^	0.18 ± 0.00^ef^	22.4 ± 1.3^gh^	2.8 ± 0.1^cd^	48.2 ± 0.4^e^	200.5 ± 3.5^c^
S31	13.1 ± 0.0^ghi^	6.1 ± 0.0^j^	2.2 ± 0.0^g^	80.2 ± 2.1^def^	3.4 ± 0.1^cde^	0.23 ± 0.03^a^	22.1 ± 1.1^gh^	3.0 ± 0.0^ab^	81.3 ± 0.1^a^	242.7 ± 0.0^a^
S40	12.9 ± 0.0^i^	6.8 ± 0.0^g^	2.8 ± 0.0^de^	84.2 ± 0.4^d^	3.5 ± 0.0^bcd^	0.18 ± 0.00^ef^	27.6 ± 0.3^c^	2.5 ± 0.0^fg^	55.6 ± 0.1^d^	172.8 ± 2.0^f^
S41	13.7 ± 0.1^bc^	6.8 ± 0.0^g^	2.4 ± 1.0^efg^	93.1 ± 1.4^bc^	3.6 ± 0.0^bc^	0.21 ± 0.01^bc^	20.0 ± 0.1^i^	2.6 ± 0.0^ef^	54.5 ± 0.3^d^	177.4 ± 0.0^ef^
S42	13.8 ± 0.2^b^	7.0 ± 0.0^ef^	2.4 ± 0.0^efg^	83.3 ± 9.9^d^	3.1 ± 0.3^fg^	0.20 ± 0.02^cd^	21.0 ± 0.5^hi^	2.6 ± 0.1^ef^	49.4 ± 0.0^e^	177.4 ± 0.1^ef^
S43	13.5 ± 0.0^cde^	6.4 ± 0.0^i^	2.2 ± 0.0^g^	98.3 ± 0.0^a^	4.0 ± 0.2^a^	0.22 ± 0.00^ab^	20.2 ± 0.5^i^	2.7 ± 0.1^de^	48.3 ± 0.1^e^	181.8 ± 0.1^de^
S14	13.2 ± 0.0^fgh^	7.6 ± 0.1^bc^	3.6 ± 0.0^ab^	75.8 ± 0.3^fg^	2.8 ± 0.0^h^	0.09 ± 0.00^kl^	19.3 ± 1.1^i^	2.5 ± 0.1^fg^	15.7 ± 0.5^l^	62.9 ± 0.0^m^
S17	13.3 ± 0.0^efg^	7.5 ± 0.0^c^	3.3 ± 0.0^bc^	76.5 ± 0.2^efg^	2.9 ± 0.0^gh^	0.10 ± 0.00^k^	29.6 ± 1.2^b^	2.6 ± 0.1^ef^	25.7 ± 2.0^i^	129.0 ± 15.9^i^
S19	13.1 ± 0.0^ghi^	7.6 ± 0.0^bc^	3.4 ± 0.0^b^	76.1 ± 0.4^efg^	2.9 ± 0.0^gh^	0.10 ± 0.00^k^	27.4 ± 1.7^c^	2.7 ± 0.0^de^	19.2 ± 2.7^k^	110.0 ± 1.2^kl^
S21	13.2 ± 0.0^fgh^	7.5 ± 0.0^c^	3.6 ± 0.1^ab^	74.8 ± 0.2^g^	2.8 ± 0.0^h^	0.08 ± 0.00^lm^	20.1 ± 1.1^i^	2.5 ± 0.1^fg^	16.0 ± 0.1^l^	63.7 ± 4.7^m^
S23	13.3 ± 0.0^efg^	7.7 ± 0.2^b^	3.6 ± 0.2^ab^	75.2 ± 0.2^fg^	2.7 ± 0.0^h^	0.08 ± 0.00^lm^	19.4 ± 0.7^i^	2.6 ± 0.2^ef^	15.8 ± 0.9^l^	66.0 ± 3.9^m^
S37	13.5 ± 0.0^cde^	8.0 ± 0.0^a^	3.5 ± 0.1^ab^	83.4 ± 0.0^d^	2.9 ± 0.0^gh^	0.10 ± 0.00^k^	33.5 ± 0.3^a^	2.3 ± 0.0^h^	22.9 ± 0.7^j^	106.1 ± 0.3^l^
S38	13.4 ± 0.0^def^	7.3 ± 0.1^d^	2.9 ± 0.1^cd^	82.7 ± 0.7^d^	3.2 ± 0.0^ef^	0.13 ± 0.00^j^	20.2 ± 0.7^i^	2.4 ± 0.0^gh^	24.0 ± 0.1^ij^	114.2 ± 0.0^k^
S39	12.9 ± 0.0^i^	7.5 ± 0.1^c^	3.3 ± 0.1^bc^	56.1 ± 1.4^h^	2.0 ± 0.1^i^	0.07 ± 0.00^m^	24.6 ± 0.6^ef^	2.5 ± 0.1^fg^	13.6 ± 0.2^l^	116.3 ± 0.9^jk^
S44	12.5 ± 0.1^j^	7.0 ± 0.1^ef^	2.8 ± 0.1^de^	84.8 ± 0.8^d^	3.4 ± 0.0^cde^	0.17 ± 0.00^fg^	22.1 ± 0.9^gh^	2.1 ± 0.1^i^	29.9 ± 0.0^h^	122.2 ± 0.1^ij^
S45	13.0 ± 0.3^hi^	7.1 ± 0.1^e^	3.9 ± 0.2^a^	75.4 ± 6.5^fg^	3.3 ± 0.3^def^	0.14 ± 0.01^ij^	26.6 ± 0.2^cd^	2.5 ± 0.0^fg^	35.9 ± 0.3^g^	146.0 ± 0.2^h^

*Mean ± standard deviation is calculated from duplicate measurements; values with different letters in the same column are significantly different at p < 0.05*.

**Table 3:** Structural parameters of rice starches for building linear regression models of structure-textural properties.

**Samples**	**Amylopectin fitting parameters**						**Amylose fitting parameters**			
	**β_Ap, 1_/0.01**	**β_Ap, 2_/0.01**	**β_Ap, 3_/0.01**	***h*_Ap, 1_/0.01**	***h*_Ap, 2_/0.01**	***h*_Ap, 3_/0.01**	**β_Am, 1_/0.001**	**β_Am, 2_/0.001**	***h*_Am, 1_/0.001**	***h*_Am, 2_/0.001**
S01	13.3 ± 0.0^hi^	6.9 ± 0.0^ef^	2.7 ± 0.0^ghi^	94.4 ± 0.0^ab^	3.6 ± 0.0^b^	0.17 ± 0.00^g^	26.7 ± 0.2^a^	3.0 ± 0.1^a^	47.1 ± 0.8^h^	177.6 ± 1.0^d^
S02	13.2 ± 0.0^ij^	6.6 ± 0.0^h^	2.3 ± 0.0^kl^	93.3 ± 0.2^bc^	3.6 ± 0.0^b^	0.21 ± 0.00^cd^	26.0 ± 0.4^ab^	3.0 ± 0.0^a^	73.7 ± 0.2^b^	187.4 ± 2.7^b^
S03	13.6 ± 0.0^ef^	7.1 ± 0.1^d^	2.9 ± 0.2^efg^	80.1 ± 0.8^gh^	2.9 ± 0.0^hi^	0.13 ± 0.00^i^	24.7 ± 2.9^b^	3.1 ± 0.0^a^	37.2 ± 0.1^i^	153.6 ± 4.3^f^
S13	12.9 ± 0.0^k^	6.9 ± 0.0^ef^	2.5 ± 0.0^ijk^	90.1 ± 1.9^bcd^	3.4 ± 0.0^cd^	0.19 ± 0.00^ef^	20.2 ± 0.5^def^	2.5 ± 0.0^cd^	48.3 ± 0.1^g^	173.6 ± 0.0^e^
S31	13.1 ± 0.0^j^	6.1 ± 0.0^j^	2.2 ± 0.0^l^	80.2 ± 2.1^gh^	3.4 ± 0.1^cd^	0.23 ± 0.03^b^	22.1 ± 1.1^c^	3.0 ± 0.0^a^	81.3 ± 0.1^a^	242.7 ± 0.0^a^
S40	12.9 ± 0.0^k^	6.8 ± 0.0^fg^	2.8 ± 0.0^fgh^	84.2 ± 0.4^efg^	3.5 ± 0.0^bc^	0.18 ± 0.00^fg^	27.6 ± 0.3^a^	2.5 ± 0.0^cd^	55.6 ± 0.1^d^	172.8 ± 2.0^e^
S41	13.7 ± 0.1^de^	6.8 ± 0.0^fg^	2.4 ± 0.1^jkl^	93.1 ± 1.4^bc^	3.6 ± 0.0^b^	0.21 ± 0.01^cd^	20.0 ± 0.1^defg^	2.6 ± 0.0^bc^	54.5 ± 0.3^e^	177.4 ± 0.0^d^
S42	13.8 ± 0.2^cd^	7.0 ± 0.0^de^	2.4 ± 0.0^jkl^	83.3 ± 9.9^efg^	3.1 ± 0.3^fg^	0.20 ± 0.02^de^	21.0 ± 0.5^cd^	2.6 ± 0.1^bc^	49.4 ± 0.0^f^	177.4 ± 0.1^d^
S43	13.5 ± 0.0^fg^	6.4 ± 0.0^i^	2.2 ± 0.0^l^	98.3 ± 0.0^a^	4.0 ± 0.2^a^	0.22 ± 0.00^bc^	20.2 ± 0.5^def^	2.7 ± 0.1^b^	48.3 ± 0.1^g^	181.8 ± 0.1^c^
S46	13.1 ± 0.1^j^	7.0 ± 0.0^de^	2.6 ± 0.2^hij^	80.1 ± 0.9^gh^	3.2 ± 0.0^ef^	0.13 ± 0.00^i^	19.8 ± 0.4^defg^	2.0 ± 0.1^gh^	30.5 ± 0.0^jk^	90.3 ± 0.0^l^
S47	13.6 ± 0.0^ef^	7.1 ± 0.2^d^	3.0 ± 0.1^ef^	89.5 ± 0.0^cd^	3.5 ± 0.1^bc^	0.15 ± 0.00^h^	15.9 ± 0.2^i^	2.2 ± 0.0^ef^	23.5 ± 0.3^m^	91.1 ± 0.1^l^
S52	13.3 ± 0.0^hi^	7.6 ± 0.0^a^	3.6 ± 0.0^ab^	87.6 ± 0.4^de^	3.3 ± 0.0^de^	0.10 ± 0.00^k^	21.5 ± 1.0^cd^	2.2 ± 0.1^ef^	12.8 ± 0.2^pq^	91.1 ± 0.1^l^
S53	13.9 ± 0.1^bc^	6.7 ± 0.2^gh^	2.4 ± 0.2^jkl^	92.5 ± 0.1^bc^	3.5 ± 0.1^bc^	0.25 ± 0.00^a^	24.6 ± 0.2^b^	2.5 ± 0.0^cd^	66.8 ± 1.1^c^	179.0 ± 7.1^cd^
S37	12.9 ± 0.0^k^	6.9 ± 0.1^ef^	2.6 ± 0.1^hij^	80.5 ± 0.7^fgh^	3.3 ± 0.0^de^	0.20 ± 0.00^de^	19.0 ± 0.4^efgh^	2.1 ± 0.1^fg^	36.6 ± 0.0^i^	81.9 ± 0.1^m^
S38	13.4 ± 0.0^gh^	7.3 ± 0.1^c^	2.9 ± 0.1^efg^	82.7 ± 0.7^fg^	3.2 ± 0.0^ef^	0.13 ± 0.00^i^	20.2 ± 0.7^def^	2.4 ± 0.0^d^	24.0 ± 0.1^m^	114.2 ± 0.0^i^
S44	12.5 ± 0.1^l^	7.0 ± 0.1^de^	2.8 ± 0.1^fgh^	84.8 ± 0.8^ef^	3.4 ± 0.0^cd^	0.17 ± 0.00^g^	22.1 ± 0.9^c^	2.1 ± 0.1^fg^	29.9 ± 0.0^k^	122.2 ± 0.1^h^
S54	13.1 ± 0.0^j^	6.1 ± 0.0^j^	2.2 ± 0.0^l^	80.2 ± 2.1^gh^	3.4 ± 0.1^cd^	0.23 ± 0.01^b^	18.9 ± 0.8^efgh^	1.9 ± 0.0^h^	24.8 ± 0.1^l^	135.0 ± 0.0^g^
S55	14.1 ± 0.0^a^	7.5 ± 0.0^ab^	3.8 ± 0.0^a^	93.2 ± 0.6^bc^	3.6 ± 0.0^b^	0.13 ± 0.01^i^	19.0 ± 1.8^efgh^	2.3 ± 0.0^e^	12.2 ± 0.2^q^	68.3 ± 0.0^n^
S56	13.5 ± 0.3^fg^	7.6 ± 0.1^a^	3.7 ± 0.4^a^	93.0 ± 3.9^bc^	3.5 ± 0.1^bc^	0.11 ± 0.01^jk^	17.8 ± 0.4^h^	2.0 ± 0.0^gh^	9.3 ± 0.1r	81.7 ± 0.0^m^
S57	13.2 ± 0.2^ij^	7.4 ± 0.1^bc^	3.1 ± 0.1^de^	76.6 ± 0.1^hi^	2.8 ± 0.0^i^	0.10 ± 0.02^k^	17.2 ± 0.2^hi^	2.5 ± 0.0^cd^	13.5 ± 1.2°*p*	95.3 ± 0.0^k^
S58	12.8 ± 0.1^k^	7.1 ± 0.1^d^	2.7 ± 0.1^ghi^	84.2 ± 0.3^efg^	3.3 ± 0.0^de^	0.17 ± 0.01^g^	20.7 ± 1.6^cde^	2.5 ± 0.1^cd^	30.8 ± 0.2^j^	116.4 ± 0.1^i^
S59	13.3 ± 0.0^hi^	7.3 ± 0.1^c^	3.3 ± 0.1^cd^	75.6 ± 0.4^i^	3.0 ± 0.0^gh^	0.12 ± 0.00^ij^	18.7 ± 1.1^fgh^	2.1 ± 0.0^fg^	13.9 ± 0.3°	81.9 ± 0.0^m^
S60	14.0 ± 0.0^ab^	7.4 ± 0.2^bc^	3.4 ± 0.0^bc^	87.3 ± 0.3^de^	3.3 ± 0.0^de^	0.12 ± 0.01^ij^	18.2 ± 0.3^gh^	2.7 ± 0.0^b^	17.9 ± 0.1^n^	99.8 ± 0.0^j^

*Mean ± standard deviation is calculated from duplicate measurements; values with different letters in the same column are significantly different at p < 0.05*.

These noticeable differences in structural properties are all desirable from the point of view of providing a wide “space” of structural parameters to test the linearity assumption, which is the aim of the present paper.

### Comparison of Thermal Properties of AWRs and DRs

**Figure 2:**
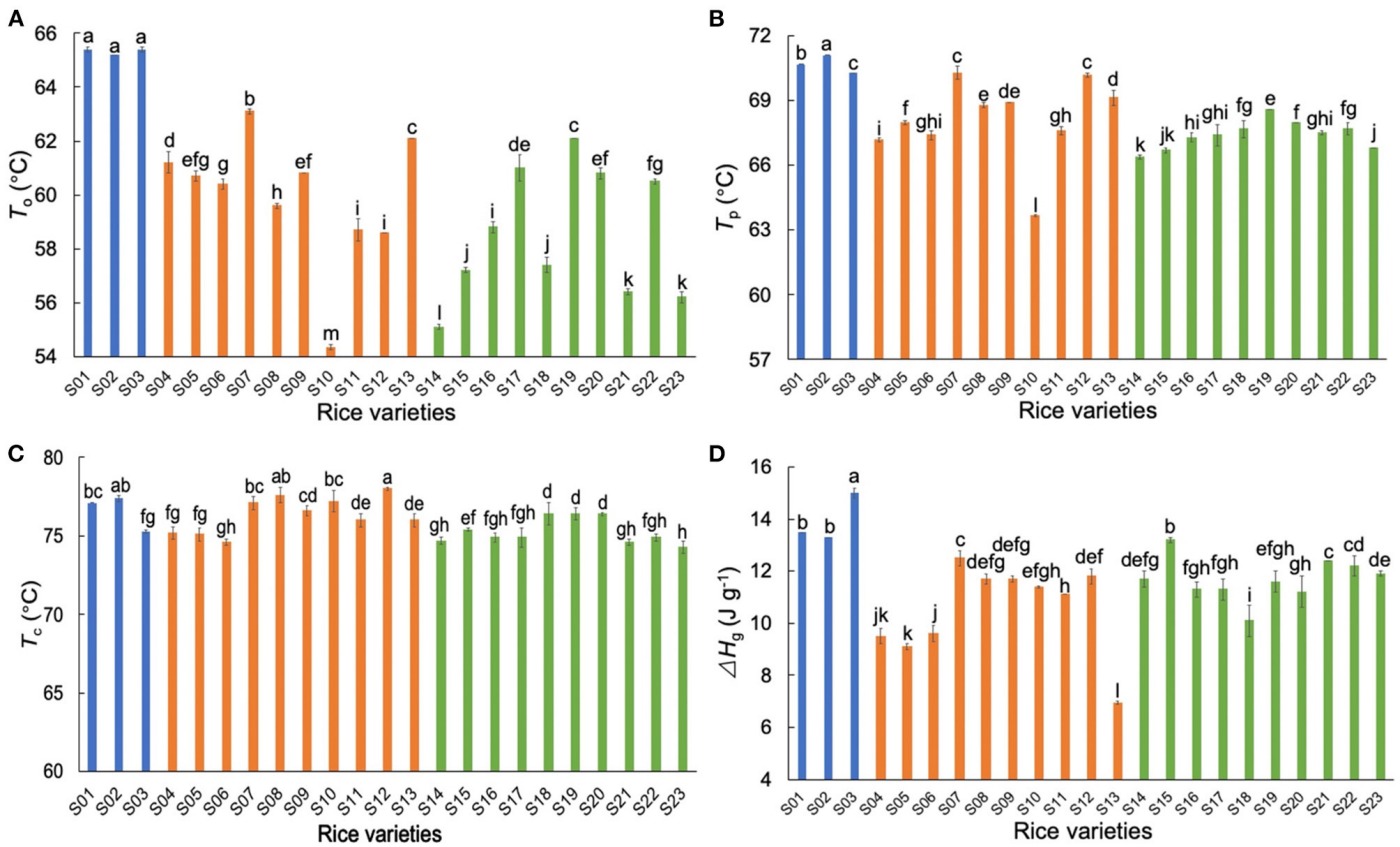
Comparison of thermal properties of native Australian Wild Rice (AWR) and Domesticated Rice (DR) starches. **(A–D)** The onset temperature (*T*_o_), the peak temperature (*T*_p_), the conclusion temperature (*T*_c_), and the crystal melting enthalpy (Δ*H*_*g*_), respectively. Blue, orange and green: Australian wild (S01–S03), *indica* (S04–S13), and *japonica* rices (S14–S23), respectively. All data were from triplicate measurements. The same letters mean no significant difference (*p* < 0.05).

The thermal data of different starches are shown in Figure 2. The trends seen here are similar to those seen elsewhere, for which explanations have been given in the literature (34–38), and thus will not be discussed further. Generally, the AWR starches had higher gelatinization temperatures (*T*_o_ and *T*_p_) than those of DR starches, showing that AWR starches had more ordered crystallinity than DR starches. The AWR starches had higher Δ*H*_g_ than those of DR starches, but the ACs of AWR starches were in the range of those of DR.

### Comparison of *in-vitro* Digestibility Properties of AWRs and DRs

**Figure 3:**
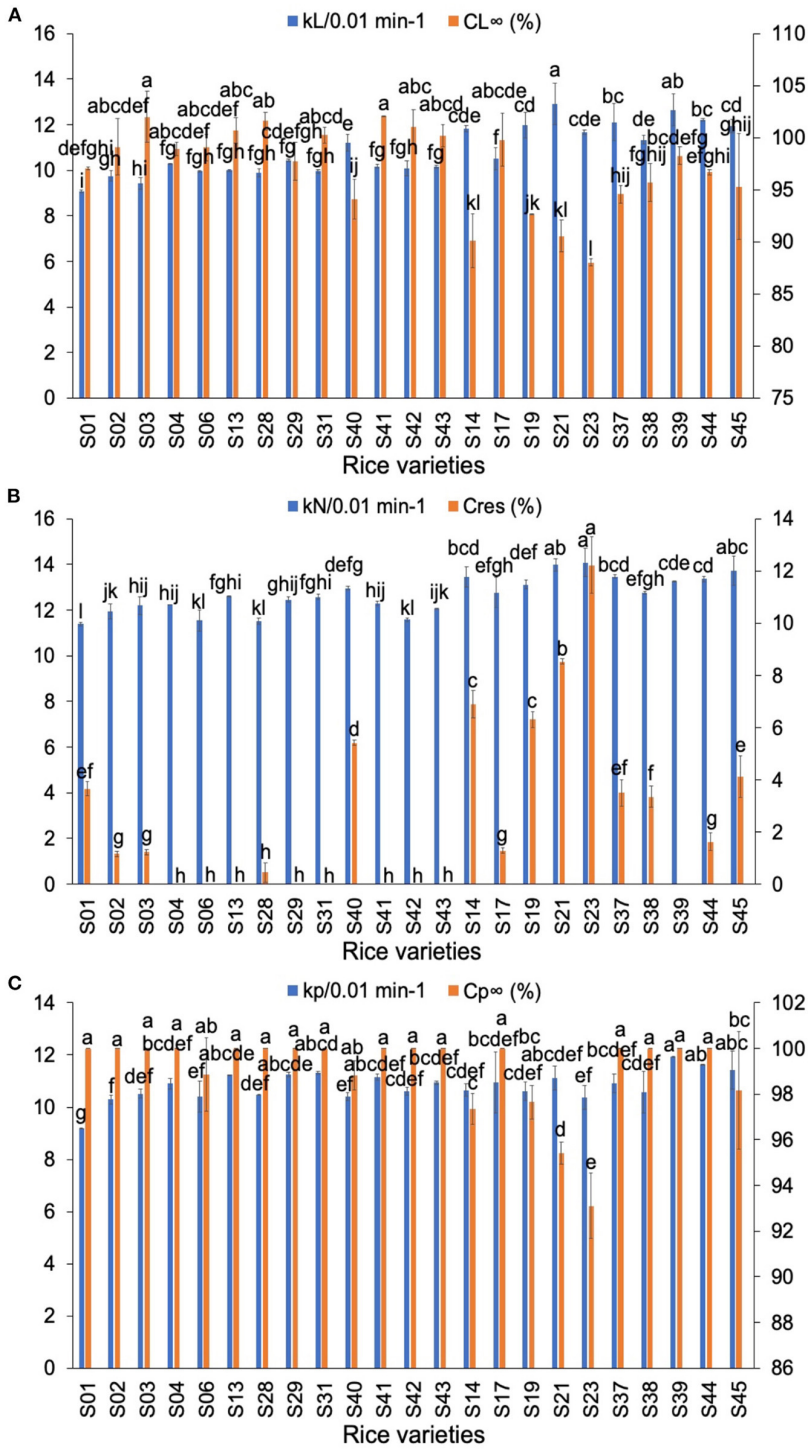
Comparison of *in-vitro* digestibility parameters of Australian Wild Rices (AWRs) and Domesticated Rices (DRs). **(A)** The digestion parameters calculated from logarithm of slopes (LoS), **(B)** the digestion parameters calculated from non-linear least-squares (NLLS), **(C)** the digestion parameters calculated from parallel models. AWRs consist of S01–S03, *indica* variety contains S04, S06, S13, S28–S29, S31, S40–S43, and *japonica* variety contains S14, S17, S19, S21, S23, S37–S39, S44–S45, respectively. *k*_L_, *k*_N_, and *k*_p_ are the digestion rate coefficients of starch of LoS, NLLS, and parallel models, respectively. *C*_L∞_ is the percentage of starch digested at very long reaction time of LoS. *C*_res_ is the fraction of residual starch (starch remaining after an extended digestion period). *C*_p∞_ is the percentage of starch digested at very long reaction time of parallel models. All data were from duplicate measurements. The same letters mean no significant difference (*p* < 0.05).

The *in-vitro* digestion curves of typical rice flours are shown in Supplementary Figure 2. Supplementary Figure 3 shows digestion data fitted to two models: a sequential model (both LoS and NLLS methods) (21) and a parallel model (22). The LoS plots shown in the Supplementary Figure 3A show that the digestion of both AWR flours and DR flours followed first-order kinetics. Similar results were obtained by Zou et al. (39). The LoS plots of rice flours were fitted with two-phase digestion kinetics (two linear regions with different slopes) (40) for different concentrations of α-amylase. The digestion rate coefficients and the fractions of starch undigested at long reaction times of all rices are shown in Figure 3. In the LoS method, *k*_L_ is the digestion rate coefficient of starch of LoS and *C*_L∞_ is the percentage of starch digested at long times. Generally, the values of *k*_L_ of AWR flours were the slowest among all samples. The AC had significant negative correlations with *k*_L_ (41) and the AC was in the order of *indica* rice varieties > AWRs > *japonica* rice varieties. This indicated that AC was the dominant but not sole factor determining *k*_L_. No obvious differences can be seen in *C*_L∞_ between AWR flours and DR flours.

In the NLLS model, the value(s) of *k*_N_ is/are the digestion rate coefficient(s) of starch of NLLS over the first-order region(s) and *C*_res_ is the fraction of residual starch (starch remaining after an extended digestion period). Generally, the *k*_N_ values were in the order of AWRs < *indica* rice varieties < *japonica* rice varieties. There were no significant differences in *C*_res_ between AWR flours and DR flours. In the parallel digestion model, *k*_p_ is the digestion rate coefficient of starch and *C*_p∞_ is the fraction of starch digested at very long reaction time. Generally, the *k*_p_ of AWR flours were slightly less than the DR counterparts. No clear differences were found in *C*_p∞_ between AWR flours and DR flours.

Generally, the AWR flours had slower digestion rate coefficients, but no noticeable differences in digestion degree, compared to DRs.

### Comparison of Textural Properties of AWRs and DRs

**Figure 4:**
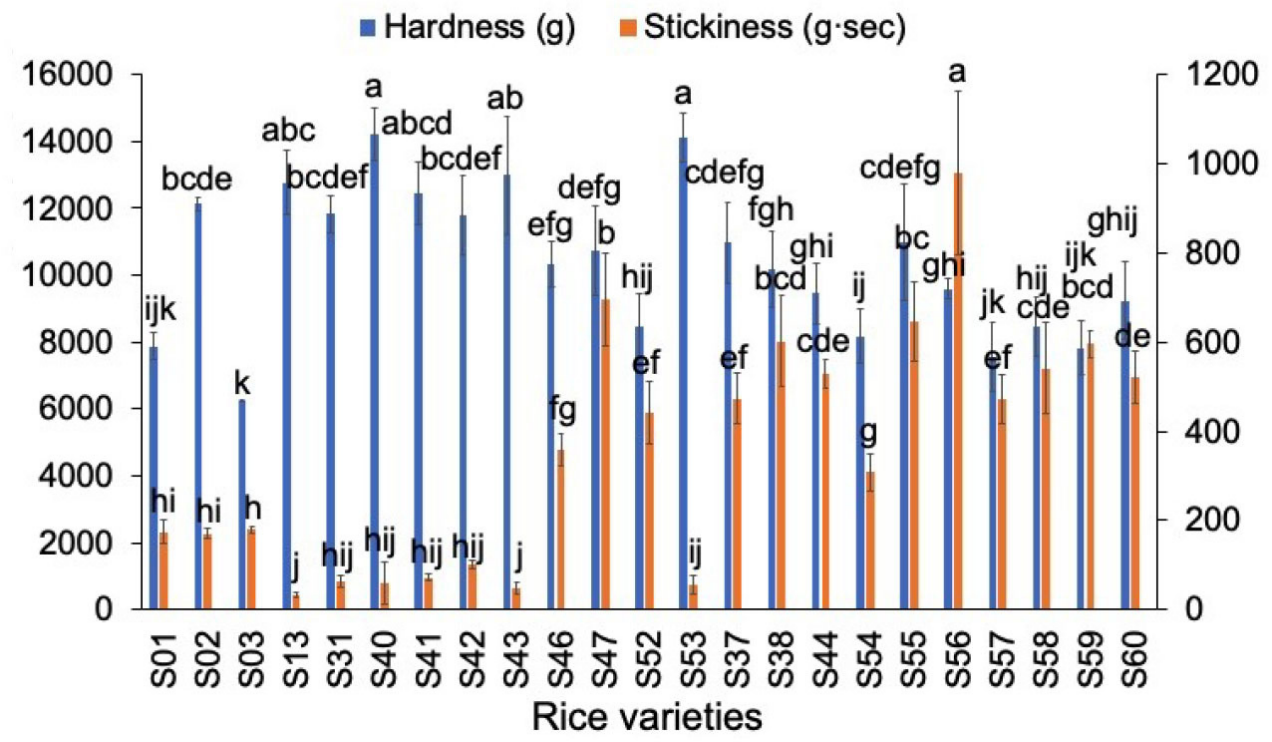
Comparison of textural parameters of Australian Wild Rices (AWRs) and Domesticated Rices (DRs). AWRs consist of S01–S03, *indica* variety contains S13, S31, S40–S43, S46–S47, S52–S53 and *japonica* variety contains S37–S38, S44, S54–S60, respectively. All data were from five times measurements. The same letters mean no significant difference (*p* < 0.05).

The texture results for different rices are shown in Figure 4. Apart from hardness and stickiness, it has been shown (42) that, for cooked rice, other attributes are not significant in texture profile analysis measurements. Therefore, only hardness and stickiness were significant as textural properties here. AWRs had neither higher nor lower hardness compared to those of DRs while AWRs had lower stickiness than those of *japonica* rices. The results are in agreement with the finding that hardness was not only affected by AC (3, 42): the hardness of AWRs with higher AC were not higher than those of *japonica* rice varieties with lower AC.

### Multiple Linear Regression Between Starch Molecular Structure and Properties

**Figure 5:**
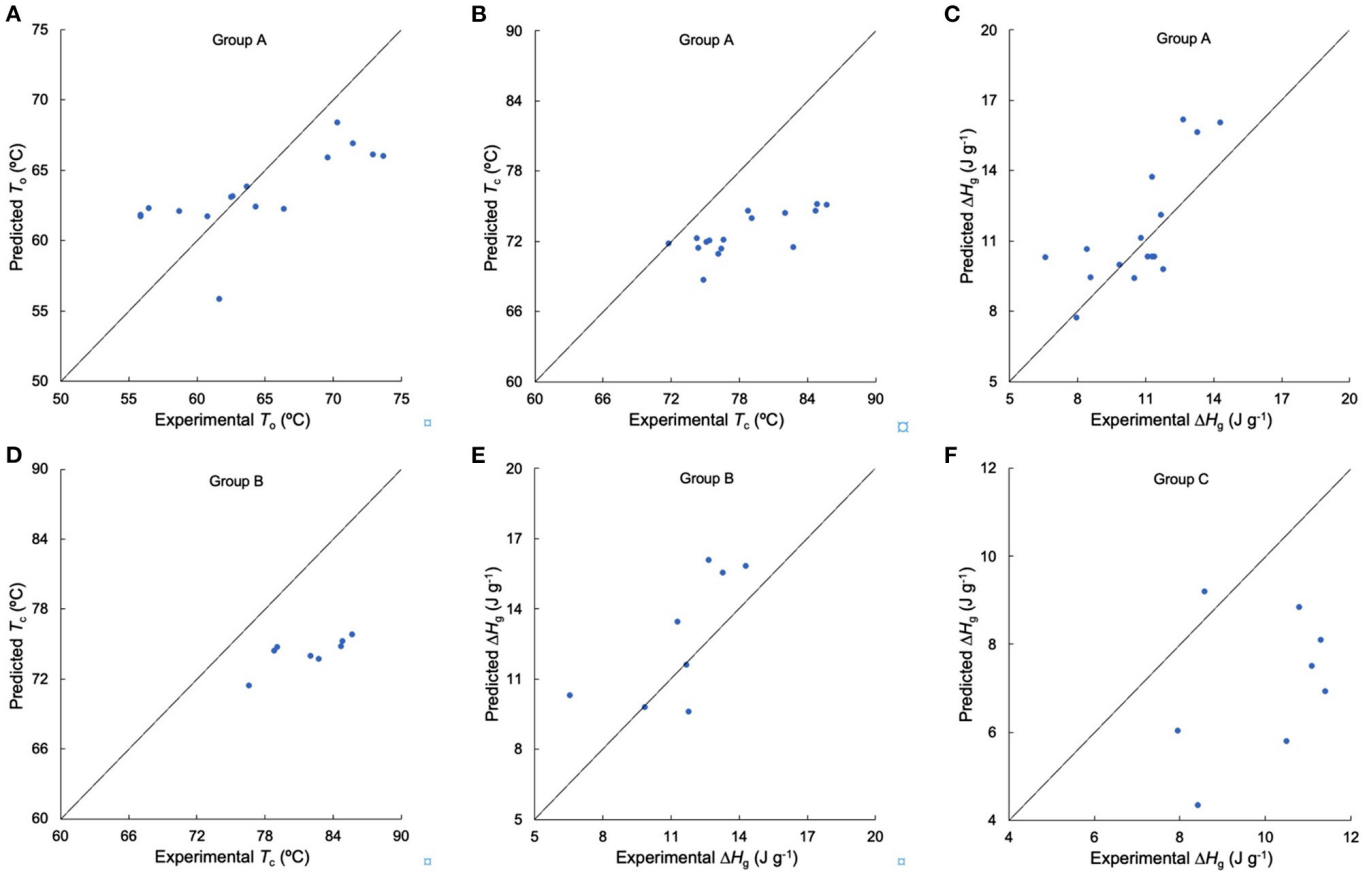
Testing the suitability of linear regression models for thermal properties. **(A–C)** Comparison of experimental and predicted *T*_o_, *T*_c_, and Δ*H*_g_ from Group A, respectively, **(D,E)** comparison of experimental and predicted *T*_c_, and Δ*H*_g_ from Group B, respectively, **(F)** comparison of experimental and predicted Δ*H*_g_ from Group C. Samples DR S24–S31 and S32–S39 were used in Group A, samples DR S24–S31 were used in Group B, and samples DR S32–S39 were used in Group C.

**Figure 6:**
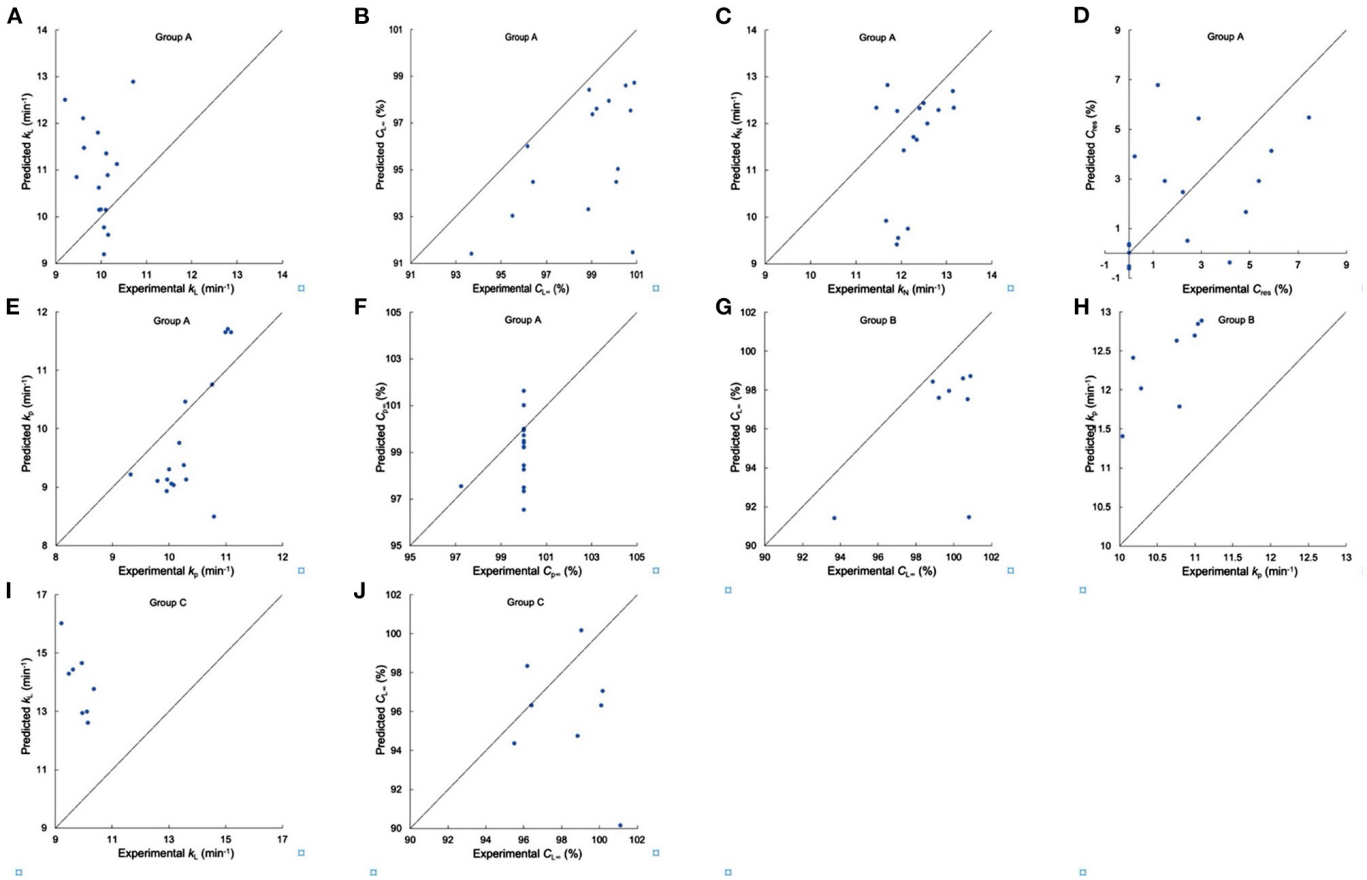
Testing the suitability of linear regression models for *in vitro* digestion properties. **(A–F)** Comparison of experimental and predicted *k*_L_, *C*__L_∞_, *k*_N_, *C*_res_, *k*_p_ and *C*_p∞_ from Group A, respectively, **(G,H)** comparison of experimental and predicted *C*__L_∞_, and *k*_p_ from Group B, respectively, **(I,J)** comparison of experimental and predicted *k*_L_ and *C*__L_∞_ from Group C. Samples DR S05, S10, S46–S51, and S18, S20, S22, S32–S36 were used in Group A, samples DR S05, S10, S46–S51 were used in Group B, and samples DR S18, S20, S22, S32–S36 were used in Group C.

**Figure 7:**
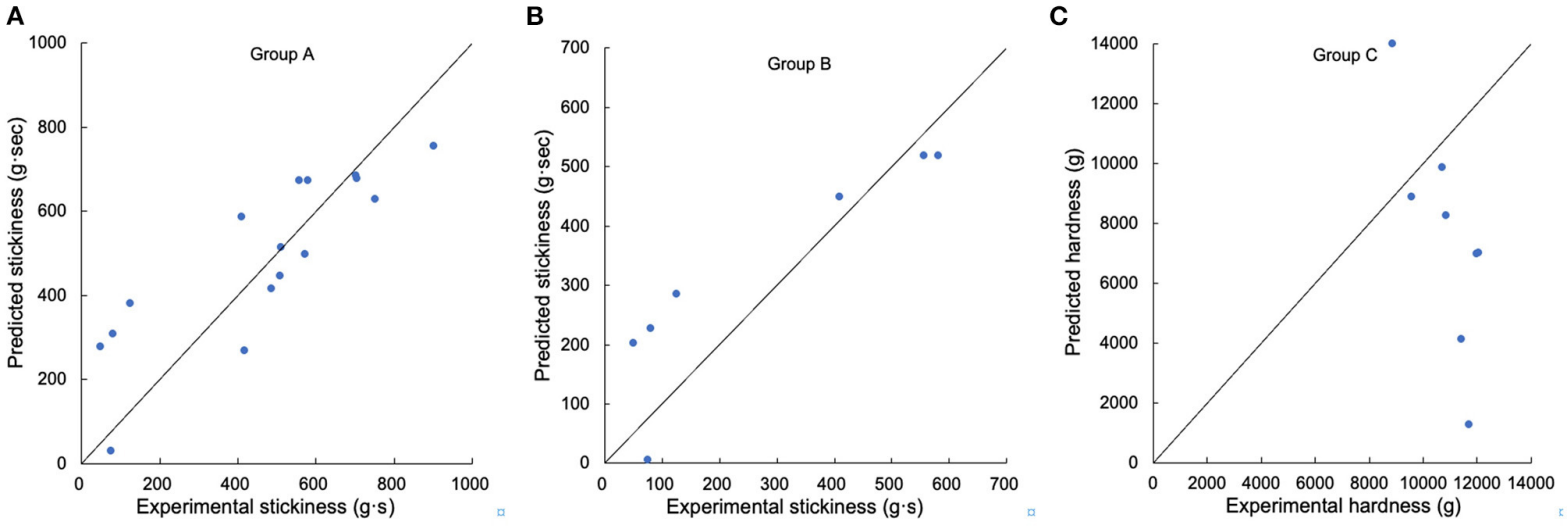
Testing the suitability of linear regression models for textural properties. **(A)** Comparison of experimental and predicted stickiness from Group A, **(B)** comparison of experimental and predicted stickiness from Group B, **(C)** comparison of experimental and predicted hardness from Group C. Samples DR S10, S61–S67, and S39, S45, S68–S73 were used in Group A, samples DR S10, S61–S67 were used in Group B, and samples DR S39, S45, S68–S73 were used in Group C.

AWRs had significant differences in structure and properties compared to those of DRs, and thus are useful for structure-property linear regressions and in testing the linearity assumption implicit in finding correlations from these. Although AC plays an important role in determining thermal, *in-vitro* digestibility and textural parameters, Am CLD fitting parameters can better explain the mechanisms of these three properties (27, 30, 31, 43). The linear regression results for rice properties based on their related starch molecular fine structure are shown in Table 4, and tests of the suitability of these linear regression models are presented in Figures 5–7. The parameters with insignificant regression coefficients are not shown.

**Table 4:** Linear regression models for rice properties.

**Functional property**	**Multiple linear regression equation**	***R* ^2^**	**RMSE**
**Thermal properties**			
Group A-*T*_o_	*T*_o_ (°C) = 39+0.3*h*_Ap, 1_	0.255*	4.44
Group A-*T*_c_	*T*_c_ (°C) = 82–3β_Ap, 1_+3β_Am, 2_+0.2*h*_Ap, 1_+2β_Ap, 3_	0.549*	6.54
Group A-Δ*H*_g_	Δ*H*_g_ (J g^−1^) = −10+10β_Am, 2_-0.04*h*_Am, 2_	0.723*	1.84
Group B-*T*_c_	*T*_c_ (°C) = 59+0.1*h*_Ap, 1_ +2β_Ap, 3_	0.738**	7.92
Group B-Δ*H*_g_	Δ*H*_g_ (J g^−1^) = −13+11β_Am, 2_-0.04*h*_Am, 2_	0.884**	2.30
Group C-Δ*H*_g_	Δ*H*_g_ (J g^−1^) = −77+0.07*h*_Ap, 1_+ 6β_Ap, 1_	0.715*	3.36
***In-vitro*** **digestion**			
Group A-*k*_L_	*k*_L_ (/0.01 min^−1^) = 21–0.04*h*_Ap, 1_-2β_Am, 2_-0.01*h*_Am, 2_	0.818*	1.48
Group A-*C*__*L*_∞_	*C*__*L*_∞_ (%) = 86+0.06*h*_Am, 2_	0.563***	4.64
Group A-*k*_N_	*k*_N_ (/0.01 min^−1^) = 16–0.07*h*_Ap, 1_+1*h*_Ap, 2_-0.01*h*_Am, 2_	0.744*	1.26
Group A-*C*_res_	*C*_res_ (%) = 10–0.05*h*_Am, 2_	0.564***	2.52
Group A-*k*_p_	*k*_p_ (/0.01 min^−1^) = 23–0.9β_Ap, 2_-0.7*h*_Ap, 2_-2β_Am, 2_	0.419*	0.92
Group A-*C*_p∞_	*C*_p∞_ (%) = 89+38*h*_Ap, 3_+0.2β_Am, 1_-0.1*h*_Am, 1_+0.03*h*_Am, 2_	0.615*	2.09
Group B-*C*__*L*_∞_	*C*__*L*_∞_ (%) = 101–0.7β_Am, 1_+ 6β_Am, 2_	0.633*	5.98
Group B-*k*_p_	*k*_p_ (/0.01 min^−1^) = 14–0.1β_Am, 1_	0.463*	1.73
Group C-*k*_L_	*k*_L_ (/0.01 min^−1^) = 19-0.1*h*_Ap, 1_+45*h*_Ap, 3_+ 0.2β_Am, 1_-0.05*h*_Am, 2_	0.883*	4.34
Group C-*C*__*L*_∞_	*C*__*L*_∞_ (%) = 85+0.08*h*_Am, 2_	0.653***	4.58
**Textural property**			
Group A-Stickiness	Stickiness (g·s) = 998–5*h*_Am, 2_	0.739***	136.38
Group B-Stickiness	Stickiness (g·s) = 779–4*h*_Am, 2_	0.693***	150.60
Group C-Hardness	Hardness (g) = 16,921+226*h*_Am, 1_-102*h*_Am, 2_-4,929β_Ap, 3_+ 8130*h*_Ap, 2_-9,8154*h*_Ap, 3_	0.890*	5551.98

*** Significant at p < 0.001; ^**^Significant at p < 0.01; ^*^Significant at p < 0.05.

The process for testing the applicability of the linearity assumption is as follows. Take Group A-Δ*H*_g_ as an example (as stated above, Group A for thermal properties consisted of samples AWR S01–S03, DR S04–S13, and S14–S23). It has been reported that Δ*H*_g_ were significantly correlated with β_Ap, 1_, β_Ap, 2_, β_Ap, 3_, *h*_Ap, 1_, β_Am, 2_ and *h*_Am, 2_ in rice starches (5, 24, 28, 34). The CLDs of these rice starches were characterized with SEC and the results were fitted to biosynthesis-based models, as discussed above. Here, we measured the thermal properties and calculated Δ*H*_g_ of these rice starches (Figure 2 and Supplementary Table 2). We obtained linear correlation coefficients between Δ*H*_g_ and its related structural parameters (β_Ap, 1_, β_Ap, 2_, β_Ap, 3_, *h*_Ap, 1_, β_Am, 2_ and *h*_Am, 2_) by a backward regression approach. We applied a linear regression model for Group A-Δ*H*_g_ to yield Δ*H*_g_ (J g^−1^) = −10+10β_Am, 2_ −0.04*h*_Am, 2_ (*p* < 0.05, *R*^2^ = 0.723, RMSE = 1.84, as shown in Table 4). If *p* > 0.05, the linear regression model would not be reliable. Finally, this linear fit was then used to predict Δ*H*_g_ values not included in the original data set (another 16 DRs, sample DR S24–S31, and S32–S39 for Group A-Δ*H*_g_) (Figure 5C). It was found that this linear model can predict Group A-Δ*H*_g_ with acceptable accuracy, because all predicted data were on or very close to the lines for experiment = predicted. If the predicted data were far away from this line, that means the linear model cannot predict the property to acceptable accuracy. The method used to build linear models between structure and property in rices can also be applicable to other cereals. The objective of the present paper is to see if linear models, commonly used to find structure-property correlations, could be used predictively. An example might be as a guide to rice breeders to try developing major changes in a structural characteristic, such as greatly increased chain lengths and hence slower digestibility. A typical case was studied; if acceptable predictability were to be found in this one case, then it would be useful to perform this simple procedure in more cases. If not, then of course the linearity assumption cannot be used predictively.

For thermal properties (Table 4), *T*_o_, and *T*_p_ had significant correlations with β_Ap, 1_, β_Ap, 2_, *h*_Ap, 1_, *h*_Ap, 2_, *h*_Am, 1_ and *h*_Am, 2_, while *T*_c_ and Δ*H*_g_ were significantly correlated with β_Ap, 1_, β_Ap, 2_, β_Ap, 3_, *h*_Ap, 1_, β_Am, 2_ and *h*_Am, 2_ (5, 24, 26–28, 30, 31, 34). In terms of the thermal properties in Group A, the linear model can predict *T*_o_ and Δ*H*_g_ with varying degrees of accuracy (Am and Ap structure accounted for 25.5 and 72.3% of the total variation of *T*_o_, and Δ*H*_g_, respectively), while the linear model cannot predict *T*_c_ to acceptable accuracy. In addition, the linear model can acceptably predict Δ*H*_g_ of Group B in terms of the CLDs of long-to-extra long Am chains (*X* > 500) (β_Am, 2_ and *h*_Am, 2_), but cannot predict that of Group C to acceptable accuracy. This might be because, as shown in Table 4, the linear regression models for Group A – Δ*H*_g_ and Group B-Δ*H*_g_ had similar structural parameters, but different from those of Group C – Δ*H*_g._ The *R*^2^ of linear regression of the *T*_c_ results for Group B was higher than that for Group A, but the number of variables in establishing linear regression of Group B was less than that of Group A. This suggests that although using similar varieties can improve the accuracy of linear regression analysis of structure-property relations, this may overlook important structural parameters, by restricting the “space” of structures. Compared to the linear regression model for gelatinization properties which confined the Am structural parameters to AC alone (6), our study considered more Am CLD structural parameters in the linear regression. The value of *h*_Ap, 1_ was the most frequent significant variable in structure-thermal relations. This is because Ap chains with DP 13–24 can form double helices (44, 45).

For *in-vitro* digestibility properties, the values of *k*_L_, *k*_N_, and *k*_p_ had significant correlations with β_Ap, 2_, *h*_Ap, 1_, *h*_Ap, 2_, *h*_Ap, 3_, β_Am, 1_, β_Am, 2_, *h*_Am, 1_, *h*_Am, 2_ and protein content, while *C*__*L*_∞_, *C*_res_, *C*_p∞_ were significantly and positively/negatively correlated with β_Ap, 2_, *h*_Ap, 3_, β_Am, 1_, β_Am, 2_, *h*_Am, 1_, *h*_Am, 2_ and protein content (5, 23, 29, 30, 32, 33). Digestibility parameters of rice flour used to test the suitability of linear regression models for *in vitro* digestion properties are shown in Supplementary Table 3. All digestion rate coefficients for Group A flours, the *k*_L_, *k*_N_, *k*_p_ values, showed slight differences among the varieties tested, which were different from the case for the digestion of pure starches (which can be ascribed to a number of reasons not discussed here because they are not relevant to the aims of the present paper) (5). Nearly all *C*_p∞_ values are 100% and *C*__*L*_∞_ values are smaller than their *C*_p∞_ counterparts. The linear model cannot predict digestion parameters of Group A to acceptable accuracy for any of the three digestion models. The values of *k*_p_ of Group B flours and the *k*_L_ values of Group C flours showed very slight differences among the varieties tested. The linear model cannot predict *C*__*L*_∞_ or *k*_p_ of Group B and *k*_L_ or *C*__*L*_∞_ of Group C to acceptable accuracy.

For texture properties, hardness had significant correlations with β_Ap, 2_, β_Ap, 3_, *h*_Ap, 2_, *h*_Ap, 3_, *h*_Am, 1_, *h*_Am, 2_ and protein content, while stickiness was significantly correlated with β_Ap, 2_, β_Ap, 3_, *h*_Ap, 2_, *h*_Ap, 3_, β_Am, 1_, *h*_Am, 1_, *h*_Am, 2_ and protein content (4, 14, 25). For Group A textural properties, the linear model can predict stickiness fairly accurately (the amount of long-to-extra long Am chains (*X* > 500) (*h*_Am, 2_) accounted for 73.9% of the total variation of stickiness). The linear regression value of *R*^2^ was always much lower than 1, but some linear regression models cannot be applied to additional samples, suggesting that the properties were controlled by more than just the CLDs, such as other structural features and food components also being important. The linear model cannot predict either Group B stickiness or Group C hardness to acceptable accuracy.

In conclusion, it is apparent that while the assumption of structure-property linearity is useful for determining statistical correlations, it is only occasionally useful for quantitative prediction of these properties. The linearity assumption is often only applicable for changes close to the conditions under which the linear coefficients are determined.

## Data Availability Statement

The original contributions presented in the study are included in the article/Supplementary Material, further inquiries can be directed to the corresponding author/s.

## Author Contributions

YZ: conceptualization, methodology, investigation, formal analysis, writing—original draft, and writing—review and editing. RH: conceptualization, supervision, and writing—review and editing. RG: conceptualization, supervision, formal analysis, and writing—review and editing. All authors contributed to the article and approved the submitted version.

## Funding

The authors acknowledge the financial support of a University of Queensland Research Training Scholarship, China Scholarship Council Scholarship and National Natural Science Foundation of China grant C1304013151101138 – a project funded by the Priority Academic Program of Jiangsu Higher Education Institutions.

## Conflict of Interest

The authors declare that the research was conducted in the absence of any commercial or financial relationships that could be construed as a potential conflict of interest.

## Publisher's Note

All claims expressed in this article are solely those of the authors and do not necessarily represent those of their affiliated organizations, or those of the publisher, the editors and the reviewers. Any product that may be evaluated in this article, or claim that may be made by its manufacturer, is not guaranteed or endorsed by the publisher.

## Abbreviations

Am: amylose
Ap: amylopectin
AWRs: Australian wild rices
DRs: domesticated rices
CLD: chain length distribution
DP: degree of polymerization
SEC: size-exclusion chromatography
LoS: logarithm of slope
NLLS: non-linear least-squares.
