# Supplementary material for: Testing the Linearity Assumption for Starch Structure-Property Relationships in Rices
Source: Front Nutr. 2022 May 23;9:916751. doi: 10.3389/fnut.2022.916751 (PMC9168890; doi:10.3389/fnut.2022.916751)
Supplement: Supplementary file 1 [file Table_1.DOCX]

Supplementary Table 1. Varieties, collection details, sub-population and chemical compositions of Australian wild rices and domesticated rices.

| Sample Name | Sample code | Sample collection details | Sub-population | Moisture content (%) | Total starch content (%)*^a^* | Total crude protein content (%)*^a^* | Amylose content (%)*^b^* | Reference |
| --- | --- | --- | --- | --- | --- | --- | --- | --- |
| S01 | *Oryza meridionalis*  (Taxa B) | North of Cairns, Queensland | wild rice | 12.9 ± 0.2^cdefgh^ | 78.0 ± 0.6 | 9.4 ± 0.2 | 23.1 ± 0.1^h^ | [present paper] |
| S02 | *Oryza officinalis* | Australian Genebank | wild rice | 12.8 ± 0.3^defghi^ | 73.4 ± 0.1 | 12.7 ± 0.0 | 24.3 ± 0.1^g^ | [present paper] |
| S03 | *Oryza australiensis* | Australian Genebank | wild rice | 12.9 ± 0.1^cdefgh^ | 82.4 ± 0.4 | 6.4 ± 0.0 | 21.0 ± 0.0^i^ | [present paper] |
| S04 | Qiguizao25 | Suzhou seed store center, China | *indica* | 13.1 ± 0.3^cdef^ | 76.4 ± 1.1 | 6.5 ± 0.2 | 25.9 ± 0.1^de^ | [present paper] |
| S05 | Yeqinglun | Suzhou seed store center, China | *indica* | 13.7 ± 0.4^ab^ | 78.6 ± 0.1 | 8.8 ± 0.5 | 24.7 ± 0.6^fg^ | [present paper] |
| S06 | Dalixiang15 | Suzhou seed store center, China | *indica* | 13.3 ± 0.2^abcd^ | 76.3 ± 0.9 | 6.8 ± 0.0 | 25.6 ± 0.3^def^ | [present paper] |
| S07 | Qianhui1385 | Suzhou seed store center, China | *indica* | 13.1 ± 0.3^cdef^ | NA | NA | 12.3 ± 0.4^pqr^ | [present paper] |
| S08 | Chang-6 | Suzhou seed store center, China | *indica* | 13.2 ± 0.3^bcde^ | NA | NA | 14.6 ± 0.8^n^ | [present paper] |
| S09 | Zhehui0506 | Suzhou seed store center, China | *indica* | 12.9 ± 0.2^cdefgh^ | NA | NA | 12.4 ± 1.2^pq^ | [present paper] |
| S10 | Basmati | PoLiu village, Lingshui County, Hainan Province, China | *indica* | 12.0 ± 0.5^kl^ | 70.15 ± 3.1 | 8.4 ± 0.2 | 25.6 ± 0.2^def^ | [present paper] |
| S11 | Shiji137 | Suzhou seed store center, China | *indica* | 13.0 ± 0.1^cdefg^ | NA | NA | 13.0 ± 0.7^op^ | [present paper] |
| S12 | Xianhui207 | Suzhou seed store center, China | *indica* | 13.3 ± 0.4^abcd^ | NA | NA | 12.1 ± 0.5^pqrs^ | [present paper] |
| S13 | MoLiZhan | Yangzhou University, Yangzhou City, Jiangsu Province, China | *indica* | 12.2 ± 0.4^jkl^ | 76.0 ± 0.1 | 8.1 ± 0.1 | 28.0 ± 0.1^bc^ | [1] |
| S14 | Jiaxuan-1250 | Suzhou seed store center, China | *japonica* | 13.1 ± 0.3^cdef^ | 84.7 ± 0.5 | 6.2 ± 0.0 | 10.8 ± 0.4^t^ | [present paper] |
| S15 | Jiaxuan-1257 | Suzhou seed store center, China | *japonica* | 13.2 ± 0.2^bcde^ | NA | NA | 8.3 ± 0.1^u^ | [present paper] |
| S16 | Xiaohongdao | Suzhou seed store center, China | *japonica* | 12.8 ± 0.3^defghi^ | NA | NA | 9.2 ± 0.0^u^ | [present paper] |
| S17 | Dalvzhong | Suzhou seed store center, China | *japonica* | 12.9 ± 0.3^cdefgh^ | NA | NA | 17.8 ± 1.2^l^ | [present paper] |
| S18 | Jinguhuang | Suzhou seed store center, China | *japonica* | 13.2 ± 0.3^bcde^ | 86.1 ± 1.6 | 6.4 ± 0.0 | 20.0 ± 1.1^j^ | [present paper] |
| S19 | Jiaxuan-1041 | Suzhou seed store center, China | *japonica* | 13.2 ± 0.3^bcde^ | NA | NA | 15.8 ± 0.0^m^ | [present paper] |
| S20 | Luohanhuang | Suzhou seed store center, China | *japonica* | 13.4 ± 0.2^abc^ | 86.0 ± 0.1 | 5.4 ± 0.0 | 19.5 ± 0.2^jk^ | [present paper] |
| S21 | Jia58zao | Suzhou seed store center, China | *japonica* | 12.7 ± 0.4^efghij^ | 85.5 ± 0.3 | 6.3 ± 0.1 | 11.1 ± 0.7^st^ | [present paper] |
| S22 | Jiaxuan-1176 | Suzhou seed store center, China | *japonica* | 13.1 ± 0.2^cdef^ | 81.6 ± 0.4 | 6.5 ± 0.0 | 11.2 ± 0.2^rst^ | [present paper] |
| S23 | Jiaxuan-1182 | Suzhou seed store center, China | *japonica* | 12.4 ± 0.3^hijk^ | 84.4 ± 0.2 | 7.2 ± 0.1 | 11.4 ± 0.0^qrst^ | [present paper] |
| S24 | Guihuazhan | Suzhou seed store center, China | *indica* | 13.1 ± 0.2^cdef^ | NA | NA | 11.3 ± 0.7^qrst^ | [present paper] |
| S25 | Jinnongsimiao | Suzhou seed store center, China | *indica* | 13.2 ± 0.1^bcde^ | NA | NA | 11.5 ± 0.2^qrst^ | [present paper] |
| S26 | Fuhui9801 | Suzhou seed store center, China | *indica* | 13.0 ± 0.5^cdefg^ | NA | NA | 13.7 ± 1.3^no^ | [present paper] |
| S27 | Minghui100 | Suzhou seed store center, China | *indica* | 13.2 ± 0.2^bcde^ | NA | NA | 8.6 ± 0.7^u^ | [present paper] |
| S28 | Gui582 | Suzhou seed store center, China | *indica* | 12.8 ± 0.2^defghi^ | 81.9 ± 0.3 | 5.6 ± 0.0 | 25.9 ± 0.3^de^ | [present paper] |
| S29 | Gui44 | Suzhou seed store center, China | *indica* | 13.8 ± 0.3^a^ | 78.8 ± 0.5 | 7.0 ± 0.1 | 24.6 ± 0.1^fg^ | [present paper] |
| S30 | Shuhui707 | Suzhou seed store center, China | *indica* | 13.3 ± 0.3^abcd^ | NA | NA | 11.8 ± 0.3^qrst^ | [present paper] |
| S31 | SanLuZhan7 | Yangzhou University, Yangzhou City, Jiangsu Province, China | *indica* | 12.8 ± 0.1^defghi^ | 74.7 ± 1.2 | 8.0 ± 0.1 | 33.2 ± 0.2^a^ | [1] |
| S32 | Jiaxuan-638 | Suzhou seed store center, China | *japonica* | 12.7 ± 0.3^efghij^ | 85.4 ± 0.3 | 6.7 ± 0.0 | 16.2 ± 0.8^m^ | [present paper] |
| S33 | Mandao | Suzhou seed store center, China | *japonica* | 13.3 ± 0.1^abcd^ | 80.2 ± 0.5 | 6.3 ± 0.0 | 23.3 ± 0.2^h^ | [present paper] |
| S34 | Putaoqing | Suzhou seed store center, China | *japonica* | 13.3 ± 0.3^abcd^ | 84.2 ± 0.0 | 5.7 ± 0.0 | 19.0 ± 0.2^jk^ | [present paper] |
| S35 | Laolaihong | Suzhou seed store center, China | *japonica* | 13.2 ± 0.2^bcde^ | 83.9 ± 0.1 | 5.7 ± 0.0 | 16.8 ± 0.7^m^ | [present paper] |
| S36 | Xiaohuangdao | Suzhou seed store center, China | *japonica* | 12.8 ± 0.2^defghi^ | 81.6 ± 0.5 | 5.2 ± 0.0 | 21.2 ± 1.5^i^ | [present paper] |
| S37 | SiDao10Hao | Yangzhou University, Yangzhou City, Jiangsu Province, China | *japonica* | 12.6 ± 0.1^fghij^ | 77.0 ± 0.1 | 6.3 ± 0.4 | 13.5 ± 0.0^o^ | [1] |
| S38 | TongGeng109 | Yangzhou University, Yangzhou City, Jiangsu Province, China | *japonica* | 12.8 ± 0.3^defghi^ | 79.8 ± 0.1 | 6.4 ± 0.2 | 19.8 ± 0.3^j^ | [1] |
| S39 | 9983 | Yangzhou University, Yangzhou City, Jiangsu Province, China | *japonica* | 12.5 ± 0.1^ghijk^ | 70.9 ± 0.9 | 6.2 ± 0.1 | 18.5 ± 0.7^kl^ | [2] |
| S40 | LongTePuB | Yangzhou University, Yangzhou City, Jiangsu Province, China | *indica* | 12.3 ± 0.2^ijk^ | 77.0 ± 0.2 | 11.2 ± 0.1 | 26.4 ± 0.1^d^ | [1] |
| S41 | TeQing | Yangzhou University, Yangzhou City, Jiangsu Province, China | *indica* | 12.5 ± 0.3^ghijk^ | 75.7 ± 0.9 | 8.1 ± 0.1 | 28.2 ± 0.9^bc^ | [1] |
| S42 | QiangO9006YouZhan | Yangzhou University, Yangzhou City, Jiangsu Province, China | *indica* | 12.7 ± 0.2^efghij^ | 77.1 ± 0.3 | 8.9 ± 0.0 | 28.7 ± 0.0^b^ | [1] |
| S43 | ZhenXian97B | Yangzhou University, Yangzhou City, Jiangsu Province, China | *indica* | 12.6 ± 0.3^fghij^ | 71.9 ± 0.2 | 10.6 ± 0.0 | 27.5 ± 0.4^c^ | [1] |
| S44 | GuangLingXiangGeng | Yangzhou University, Yangzhou City, Jiangsu Province, China | *japonica* | 12.5 ± 0.3^ghijk^ | 78.0 ± 0.6 | 7.1 ± 0.0 | 21.5 ± 0.9^i^ | [1] |
| S45 | R254 | Yangzhou University, Yangzhou City, Jiangsu Province, China | *japonica* | 12.7 ± 0.1^efghij^ | 82.0 ± 0.1 | 7.1 ± 0.4 | 21.8 ± 1.5^i^ | [2] |
| S46 | GuoDao6 | Yangzhou University, Yangzhou City, Jiangsu Province, China | *indica* | 12.3 ± 0.0^ijk^ | 80.8 ± 0.4 | 7.7 ± 0.4 | 19.2 ± 0.1^jk^ | [1] |
| S47 | CV17 | Yangzhou University, Yangzhou City, Jiangsu Province, China | *indica* | 12.4 ± 0.2^hijk^ | 78.4 ± 0.4 | 6.9 ± 0.1 | 19.7 ± 0.4^j^ | [1] |
| S48 | PACHCHAIPERUMAL 2462-11::IRGC 3474-1 | PoLiu village, Lingshui County, Hainan Province, China | *indica* | 11.8 ± 0.4^l^ | 79.3 ± 0.5 | 7.5 ± 0.0 | 25.2 ± 0.1^efg^ | [3] |
| S49 | RACE PERUMAL::IRGC 55347-1 | PoLiu village, Lingshui County, Hainan Province, China | *indica* | 11.0 ± 0.1^m^ | 80.0 ± 1.4 | 8.3 ± 0.1 | 25.5 ± 0.2^def^ | [3] |
| S50 | FL 478 | PoLiu village, Lingshui County, Hainan Province, China | *indica* | 13.0 ± 0.3^cdefg^ | 78.9 ± 0.4 | 12.9 ± 0.1 | 26.5 ± 0.4^d^ | [3] |
| S51 | IRGA 318-11-9-2A::IRGC 117340-1 | PoLiu village, Lingshui County, Hainan Province, China | *indica* | 13.0 ± 0.2^cdefg^ | 79.4 ± 0.8 | 8.4 ± 0.1 | 24.4 ± 0.2^g^ | [3] |
| S52 | MingHui63 | Yangzhou University, Yangzhou City, Jiangsu Province, China | *indica* | 12.5 ± 0.1^ghijk^ | NA | 8.0 ± 0.2 | 16.3 ± 0.2^m^ | [1] |
| S53 | MaWeiZhan | Yangzhou University, Yangzhou City, Jiangsu Province, China | *indica* | 12.4 ± 0.2^hijk^ | NA | 11.9 ± 0.1 | 29.2 ± 1.0^b^ | [1] |
| S54 | TaiZhou0206 | Yangzhou University, Yangzhou City, Jiangsu Province, China | *japonica* | 12.4 ± 0.3^hijk^ | NA | 6.2 ± 0.4 | 23.8 ± 0.1^h^ | [1] |
| S55 | HuaNuo | Yangzhou University, Yangzhou City, Jiangsu Province, China | *japonica* | 12.5 ± 0.1^ghijk^ | NA | 8.5 ± 0.0 | 14.8 ± 1.4^n^ | [1] |
| S56 | QingluZhan11 | Yangzhou University, Yangzhou City, Jiangsu Province, China | *japonica* | 12.6 ± 0.1^fghij^ | NA | 7.5 ± 0.2 | 16.8 ± 0.1^m^ | [1] |
| S57 | XiangGeng111/C9083 | Yangzhou University, Yangzhou City, Jiangsu Province, China | *japonica* | 12.4 ± 0.2^hijk^ | NA | 7.4 ± 0.1 | 16.1 ± 0.2^m^ | [1] |
| S58 | XiangGeng49 | Yangzhou University, Yangzhou City, Jiangsu Province, China | *japonica* | 12.7 ± 0.1^efghij^ | NA | 8.4 ± 0.1 | 19.8 ± 0.5^j^ | [1] |
| S59 | Yu44 | Yangzhou University, Yangzhou City, Jiangsu Province, China | *japonica* | 12.2 ± 0.4^jkl^ | NA | 7.2 ± 0.1 | 16.7 ± 1.2^m^ | [1] |
| S60 | ZhenDao99 | Yangzhou University, Yangzhou City, Jiangsu Province, China | *japonica* | 12.0 ± 0.2^kl^ | NA | 7.0 ± 0.1 | 18.9 ± 0.1^jk^ | [1] |
| S61 | SJR3611 | Yangzhou University, Yangzhou City, Jiangsu Province, China | *indica* | 12.0 ± 0.1^kl^ | NA | 6.5 ± 0.1 | 14.4 ± 0.3^n^ | [1] |
| S62 | YanHui559 | Yangzhou University, Yangzhou City, Jiangsu Province, China | *indica* | 12.3 ± 0.0^ijk^ | NA | 6.8 ± 0.0 | 14.2 ± 0.5^n^ | [1] |
| S63 | GIZA 178::GERVEX 1681-C1 | PoLiu village, Lingshui County, Hainan Province, China | *indica* | 12.4 ± 0.2^hijk^ | NA | 8.9 ± 0.2 | 10.5 ± 0.1^t^ | [4] |
| S64 | IRRI 146::G1 | PoLiu village, Lingshui County, Hainan Province, China | *indica* | 12.0 ± 0.1^kl^ | NA | 7.5 ± 0.1 | 18.3 ± 0.1^kl^ | [4] |
| S65 | KABERI::IRGC 66801-1 | PoLiu village, Lingshui County, Hainan Province, China | *indica* | 12.5 ± 0.1^ghijk^ | NA | 11.2 ± 0.1 | 24.6 ± 0.0^fg^ | [4] |
| S66 | LvHuangzan | PoLiu village, Lingshui County, Hainan Province, China | *indica* | 12.2 ± 0.4^jkl^ | NA | 9.7 ± 0.0 | 24.1 ± 0.4^g^ | [4] |
| S67 | GuiZhao2 | PoLiu village, Lingshui County, Hainan Province, China | *indica* | 12.6 ± 0.1^fghij^ | NA | 8.7 ± 0.1 | 25.1 ± 0.2^efg^ | [4] |
| S68 | CR-140 | Yangzhou University, Yangzhou City, Jiangsu Province, China | *japonica* | 12.3 ± 0.2^ijk^ | NA | 9.8 ± 0.0 | 13.3 ± 0.2^o^ | [1] |
| S69 | SJR6783-1 | Yangzhou University, Yangzhou City, Jiangsu Province, China | *japonica* | 12.2 ± 0.4^jkl^ | NA | 8.0 ± 0.2 | 14.2 ± 0.2^n^ | [1] |
| S70 | SWR22 | Yangzhou University, Yangzhou City, Jiangsu Province, China | *japonica* | 12.4 ± 0.2^hijk^ | NA | 7.9 ± 0.2 | 19.1 ± 0.4^j^ | [1] |
| S71 | Ballila | Yangzhou University, Yangzhou City, Jiangsu Province, China | *japonica* | 12.2 ± 0.4^jkl^ | NA | 7.8 ± 0.1 | 21.7 ± 0.7^i^ | [1] |
| S72 | YanGeng5Hao | Yangzhou University, Yangzhou City, Jiangsu Province, China | *japonica* | 12.6 ± 0.1^fghij^ | NA | 11.3 ± 0.0 | 16.3 ± 1.5^m^ | [1] |
| S73 | HP121 | Yangzhou University, Yangzhou City, Jiangsu Province, China | *japonica* | 12.2 ± 0.4^jkl^ | NA | 9.8 ± 0.1 | 12.8 ± 0.7^op^ | [2] |

*^a^* Data expressed on a dry basis;

*^b^* Data expressed on a basis of total starch; values are the means of duplicate ± SD. Means followed by the same letters did not differ significantly (*p* < 0.05).

*^c^* NA: Not applicable.

*^d^* Chemical compositions of domesticated rices (DRs) obtained under the same or similar conditions as Australian wild rices (AWRs) are retrieved from the literature.

^e^ [1] Li, C.; Ji, Y.; Li, E. Understanding the influences of rice starch fine structure and protein content on cooked rice texture. *Starch‐Stärke*, 2100253; [2] measured in author’s group by Enpeng Li and co-workers; [3] Zhu, J., Yu, W., Zhang, C., Zhu, Y., Xu, J., Li, E., Gilbert, R. G., & Liu, Q. (2019). Carbohydrate Polymers, 230, 115656; [4] measured in author’s group by Cheng Li and co-workers.

Supplementary Table 2. Gelatinization properties of rice starches used to test the suitability of linear regression models for thermal properties

| Sample Name | *T*_o_ (ºC) | *T*_p_ (ºC) | *T*_c_ (ºC) | Δ*H*_g_ (J g^–1^) |
| --- | --- | --- | --- | --- |
| S24 | 66.4 ± 0.3^f^ | 71.7 ± 0.1^e^ | 79.1 ± 0.3^e^ | 11.7 ± 0.2^de^ |
| S25 | 64.3 ± 0.1^g^ | 70.2 ± 0.1^f^ | 78.8 ± 0.8^e^ | 11.3 ± 0.1^f^ |
| S26 | 69.6 ± 0.0^e^ | 78.3 ± 0.1^c^ | 84.7 ± 0.1^b^ | 13.3 ± 0.1^b^ |
| S27 | 73.7 ± 0.1^a^ | 80.1 ± 0.1^a^ | 85.7 ± 0.1^a^ | 14.3 ± 0.0^a^ |
| S28 | 71.5 ± 0.2^c^ | 76.1 ± 0.3^d^ | 82.8 ± 0.2^c^ | 11.8 ± 0.3^d^ |
| S29 | 70.3 ± 0.4^d^ | 76.3 ± 0.2^d^ | 82.0 ± 0.4^d^ | 9.9 ± 0.1^i^ |
| S30 | 72.9 ± 0.2^b^ | 78.7 ± 0.3^b^ | 84.9 ± 0.4^b^ | 12.7 ± 0.1^c^ |
| S31 | 62.6 ± 0.4^i^ | 69.4 ± 0.1^h^ | 76.6 ± 0.1^f^ | 6.6 ± 0.1^l^ |
| S32 | 60.8 ± 0.3^k^ | 67.6 ± 0.3^j^ | 74.3 ± 0.4^j^ | 11.1 ± 0.3^fg^ |
| S33 | 58.7 ± 0.1^l^ | 64.7 ± 0.0^m^ | 71.8 ± 0.0^k^ | 10.5 ± 0.3^h^ |
| S34 | 55.9 ± 0.8^n^ | 66.9 ± 0.2^k^ | 74.4 ± 0.1^ij^ | 11.3 ± 0.2^f^ |
| S35 | 56.5 ± 0.3^m^ | 67.1 ± 0.1^k^ | 75.1 ± 0.1^gh^ | 10.8 ± 0.0^gh^ |
| S36 | 55.9 ± 0.0^n^ | 66.2 ± 0.3^l^ | 75.4 ± 0.1^g^ | 11.4 ± 0.2^ef^ |
| S37 | 62.7 ± 0.2^i^ | 69.2 ± 0.2^h^ | 76.2 ± 0.1^f^ | 8.0 ± 0.1^k^ |
| S38 | 63.7 ± 0.3^h^ | 69.9 ± 0.1^g^ | 76.5 ± 0.2^f^ | 8.6 ± 0.3^j^ |
| S39 | 61.7 ± 0.1^j^ | 68.1 ± 0.0^i^ | 74.9 ± 0.4^hi^ | 8.4 ± 0.3^j^ |

All data were from triplicate measurements. The same letters in the same column mean no significant difference (*p* < 0.05).

Supplementary Table 3. Digestibility parameters of rice flour used to test the suitability of linear regression models for *in vitro* digestion properties

| Sample Name | Sequential model | | | | Parallel model | |
| --- | --- | --- | --- | --- | --- | --- |
|  | LoS | | NLLS | |  |  |
|  | *k*_L_/0.01 min^-1^ | *C*_L∞_ (%) | *k*_N_/0.01 min^-1^ | *C*_res_ (%) | *k*_p_/0.01 min^-1^ | *C*_p∞_ (%) |
| S05 | 10.1 ± 0.1^abcd^ | 98.9 ± 0.9^abc^ | 12.3 ± 0.0^bcd^ | 4.2 ± 0.0^e^ | 10.0 ± 0.0^bc^ | 100.0 ± 0.0^a^ |
| S10 | 10.2 ± 0.2^abc^ | 99.2 ± 2.0^ab^ | 12.1 ± 0.1^cdef^ | 0.0 ± 0.0^i^ | 10.8 ± 0.1^a^ | 100.0 ± 0.0^a^ |
| S46 | 10.7 ± 0.3^a^ | 93.7 ± 1.2^d^ | 13.1 ± 0.3^a^ | 7.4 ± 0.2^a^ | 10.3 ± 0.4^b^ | 97.2 ± 0.7^b^ |
| S47 | 9.6 ± 0.5^cde^ | 100.8 ± 0.6^a^ | 12.4 ± 0.7^bcd^ | 2.9 ± 0.3^f^ | 10.2 ± 0.5^bc^ | 100.0 ± 0.0^a^ |
| S48 | 10.1 ± 0.3^abcd^ | 99.8 ± 1.5^a^ | 11.9 ± 0.1^def^ | 0.0 ± 0.0^i^ | 11.0 ± 0.1^a^ | 100.0 ± 0.0^a^ |
| S49 | 10.0 ± 0.3^bcd^ | 100.5 ± 2.8^a^ | 11.9 ± 0.1^def^ | 0.0 ± 0.0^i^ | 11.0 ± 0.2^a^ | 100.0 ± 0.0^a^ |
| S50 | 10.0 ± 0.2^abcd^ | 100.7 ± 1.4^a^ | 12.1 ± 0.1^cde^ | 0.0 ± 0.0^i^ | 11.1 ± 0.0^a^ | 100.0 ± 0.0^a^ |
| S51 | 10.1 ± 0.2^abcd^ | 100.9 ± 1.1^a^ | 11.7 ± 0.0^ef^ | 0.0 ± 0.0^i^ | 10.8 ± 0.0^a^ | 100.0 ± 0.0^a^ |
| S18 | 9.5 ± 0.4^de^ | 100.2 ± 1.2^a^ | 11.9 ± 0.1^def^ | 2.2 ± 0.4^g^ | 10.0 ± 0.0^bc^ | 100.0 ± 0.0^a^ |
| S20 | 10.4 ± 0.1^ab^ | 96.4 ± 1.3^bcd^ | 13.2 ± 0.5^a^ | 5.4 ± 0.2^c^ | 10.0 ± 0.3^bc^ | 100.0 ± 0.0^a^ |
| S22 | 9.2 ± 0.8^e^ | 101.1 ± 2.0^a^ | 11.7 ± 0.6^ef^ | 1.2 ± 0.3^h^ | 10.0 ± 0.2^bc^ | 100.0 ± 0.0^a^ |
| S32 | 9.9 ± 0.1^bcd^ | 95.5 ± 1.2^d^ | 12.5 ± 0.2^bcd^ | 5.9 ± 0.1^b^ | 9.3 ± 0.1^d^ | 100.0 ± 0.0^a^ |
| S33 | 10.0 ± 0.0^bcd^ | 99.0 ± 0.4^abc^ | 12.3 ± 0.1^bcde^ | 2.4 ± 0.1^g^ | 10.3 ± 0.1^b^ | 100.0 ± 0.0^a^ |
| S34 | 10.1 ± 0.5^abcd^ | 100.1 ± 2.3^a^ | 12.8 ± 0.5^ab^ | 1.5 ± 0.1^h^ | 10.3 ± 0.5^b^ | 100.0 ± 0.0^a^ |
| S35 | 9.6 ± 0.3^cde^ | 98.9 ± 2.1^abc^ | 11.5 ± 0.2^f^ | 0.2 ± 0.3^i^ | 10.1 ± 0.1^bc^ | 100.0 ± 0.0^a^ |
| S36 | 10.1 ± 0.3^abc^ | 96.2 ± 1.5^cd^ | 12.6 ± 0.3^abc^ | 4.8 ± 0.1^d^ | 9.8 ± 0.2^c^ | 100.0 ± 0.0^a^ |

*^a^ k*_L,_ *k*_N,_ and *k*_p_ are the digestion rate coefficients of starch of logarithm of slopes (LoS), non-linear least-squares (NLLS), and parallel models, respectively. *C*_L∞_ is the percentage of starch digested at very long reaction time of LoS. *C*_res_ is the fraction of residual starch (starch remaining after an extended digestion period). *C*_p∞_ is the percentage of starch digested at very long reaction time of parallel models. *^b^* All data were from duplicate measurements. The same letters mean in the same column no significant difference (*p* < 0.05).


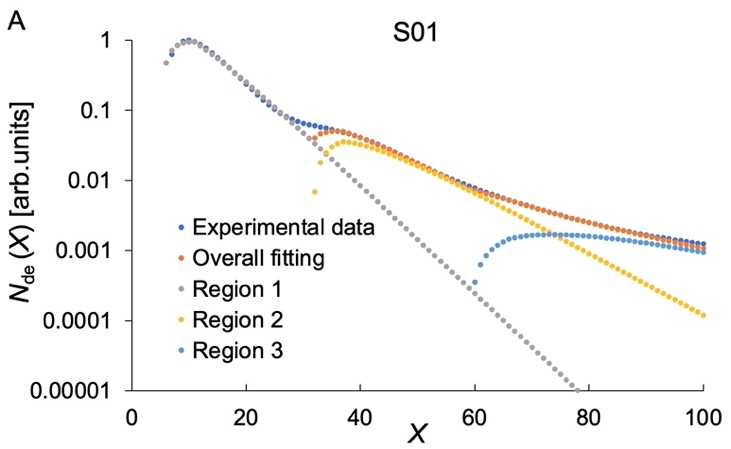

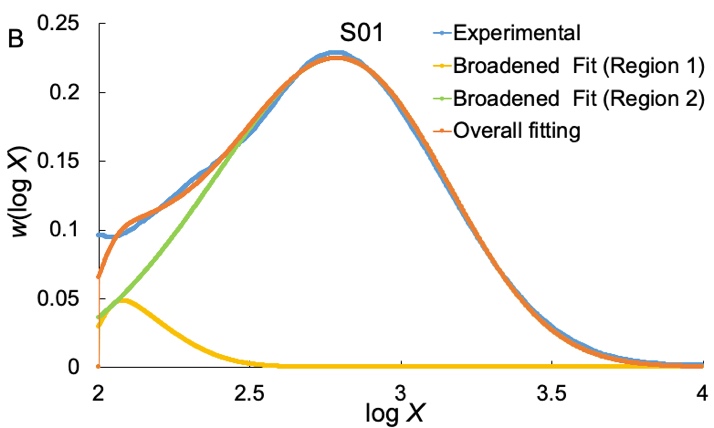


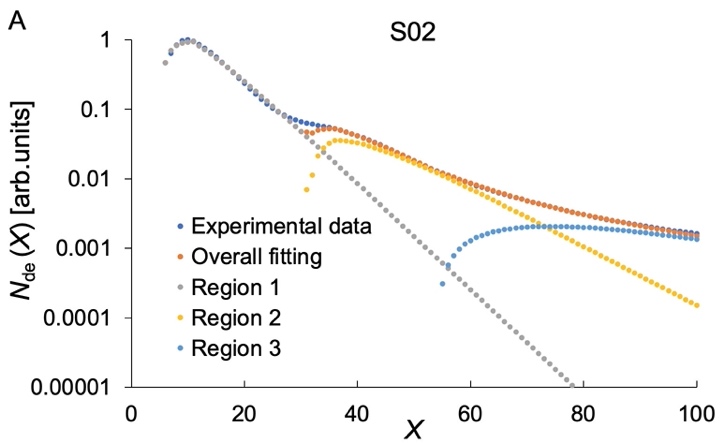

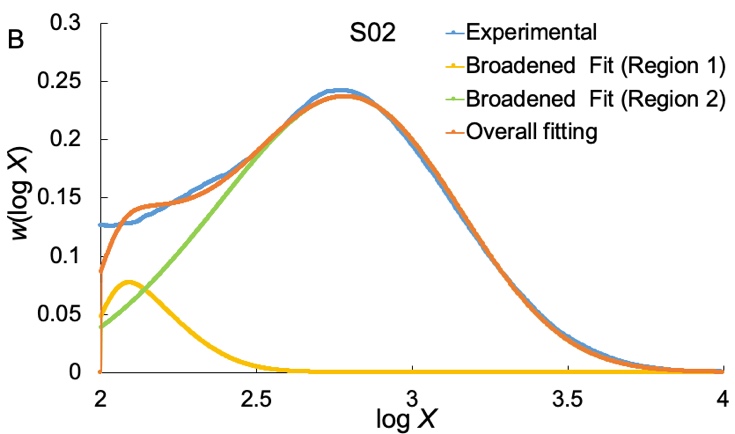


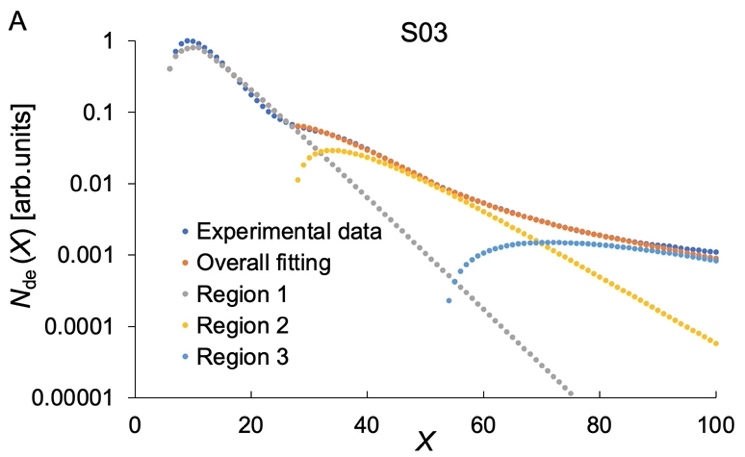

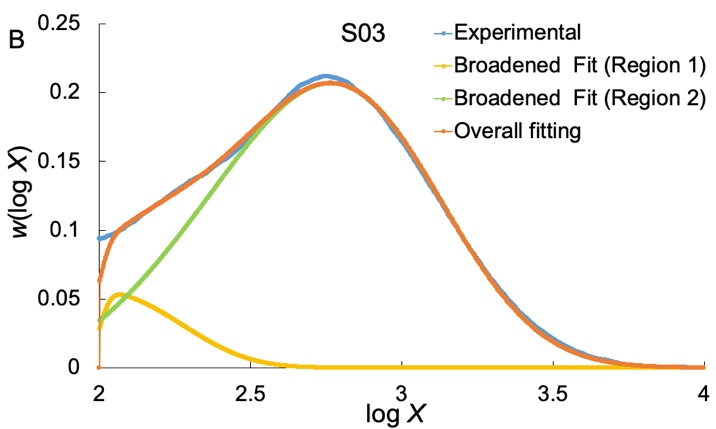


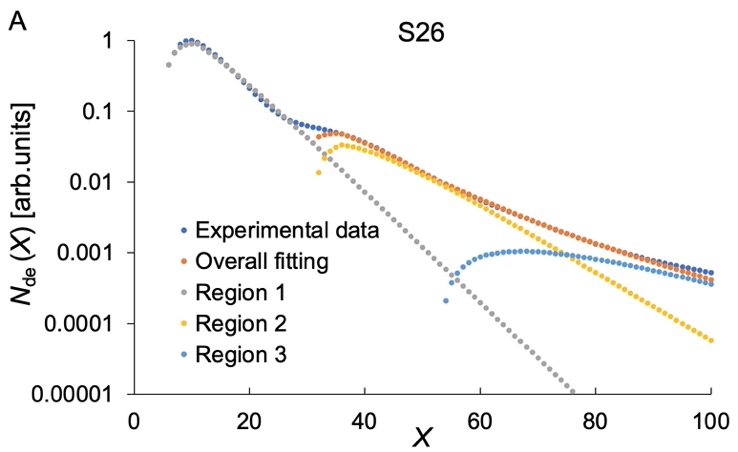

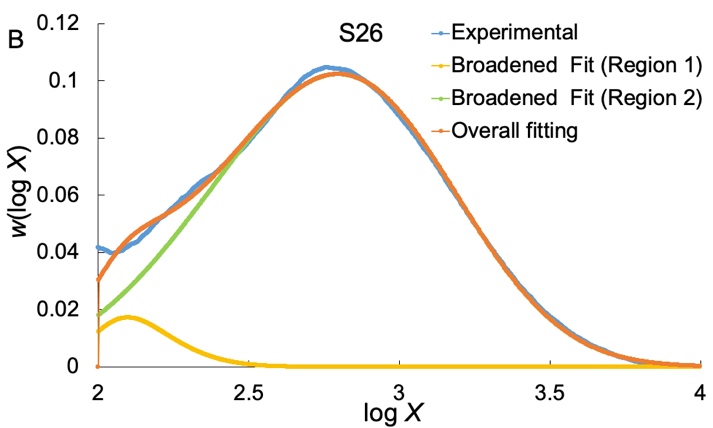


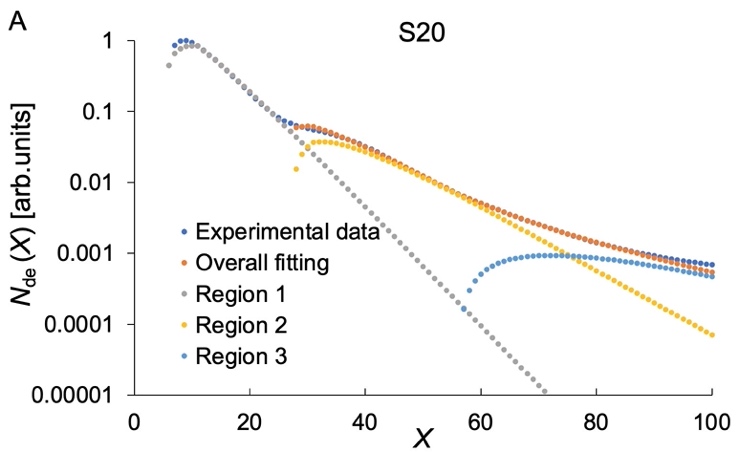

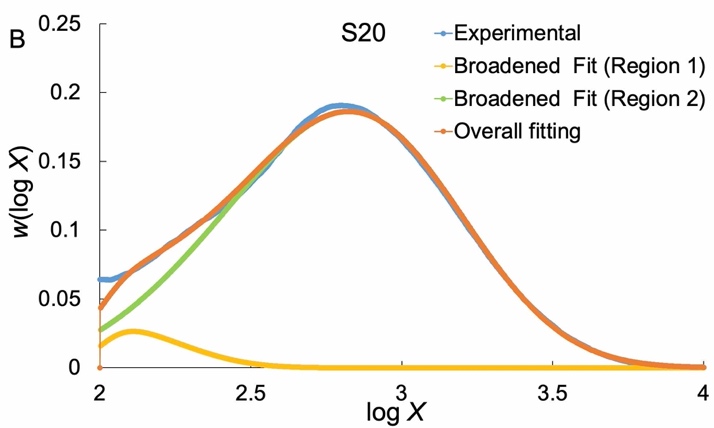


Supplementary Figure 1. Fitting results of amylopectin (A) and amylose (B) CLD for AWR (S01-S03) and DRs (*indica* rice: S26; *japonica* rice: S20). Fitting results of amylopectin and amylose CLD for both S26 and S20 were analysed with the same conditions as for AWRs.


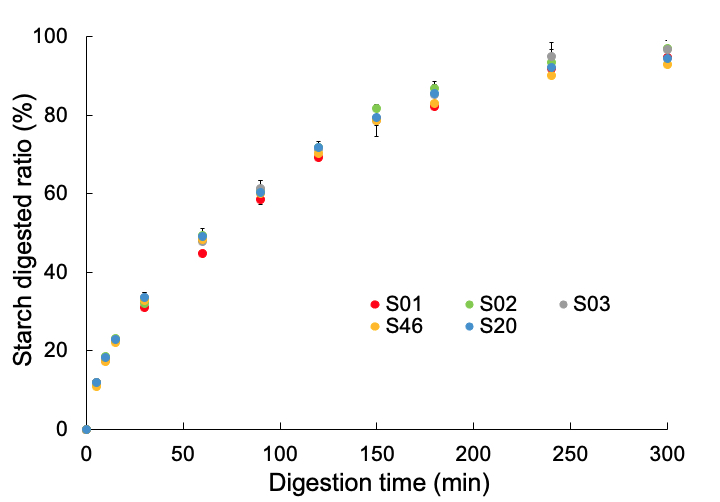


Supplementary Figure 2. Typical starch digestion curves for cooked milled AWRs (S01-S03) and DRs (*indica* rice: S46; *japonica* rice: S20).


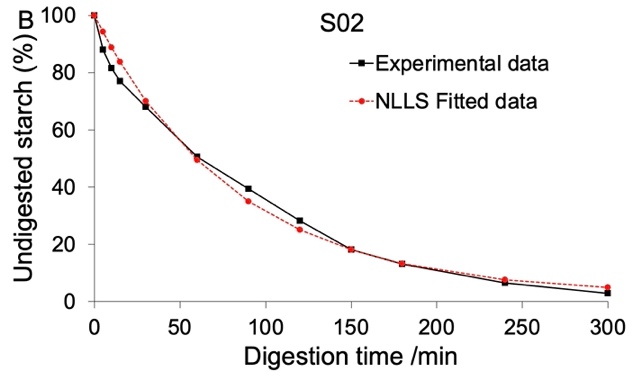

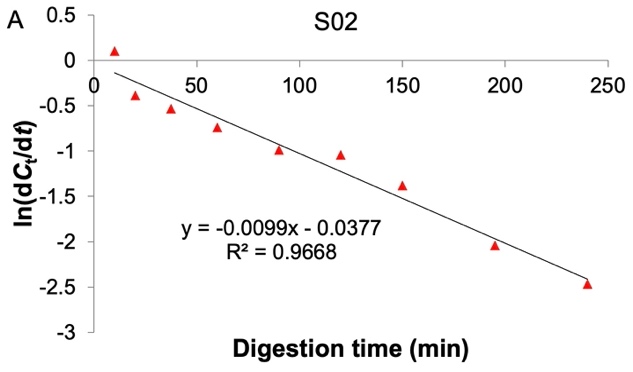

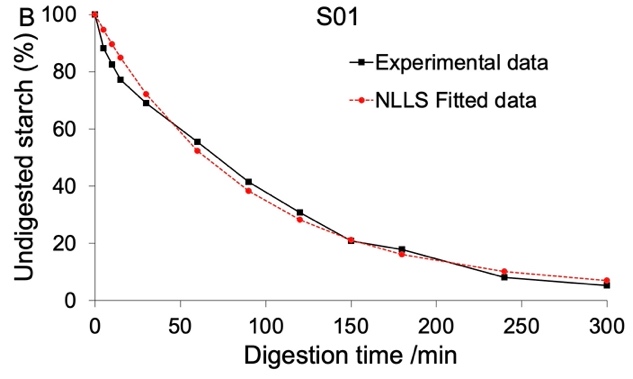

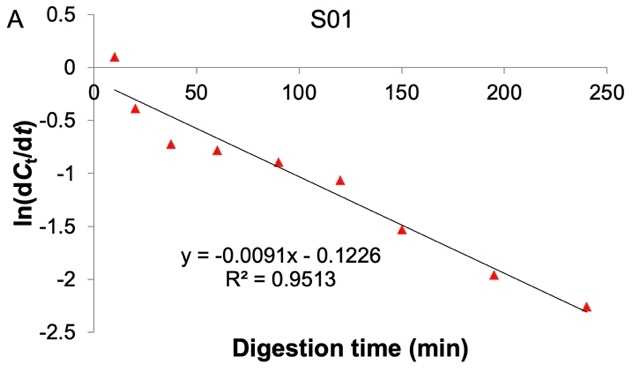

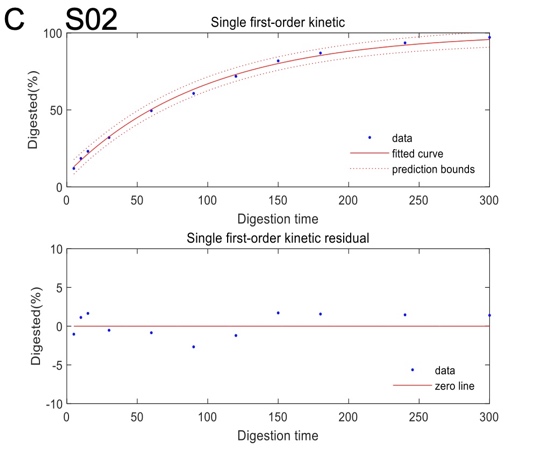

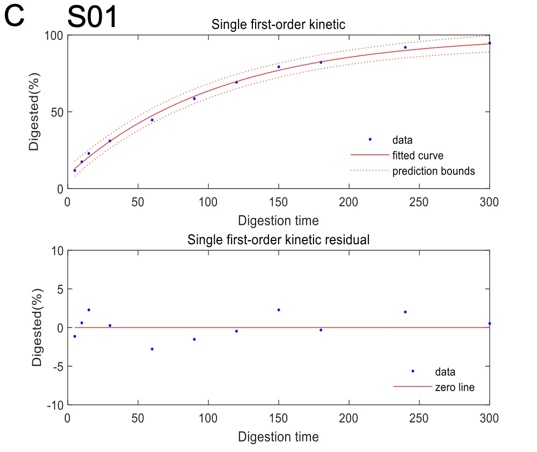

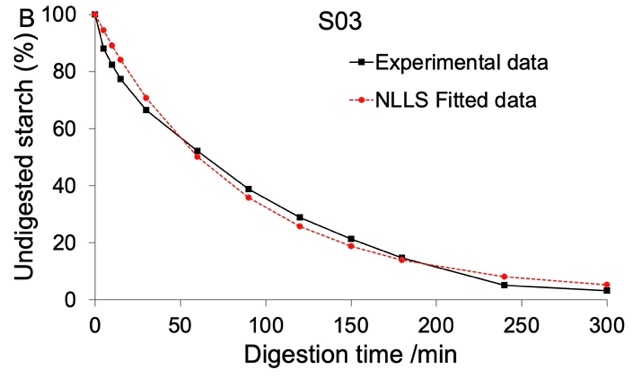

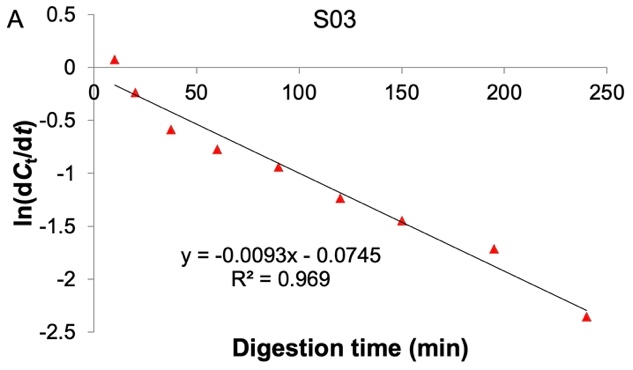

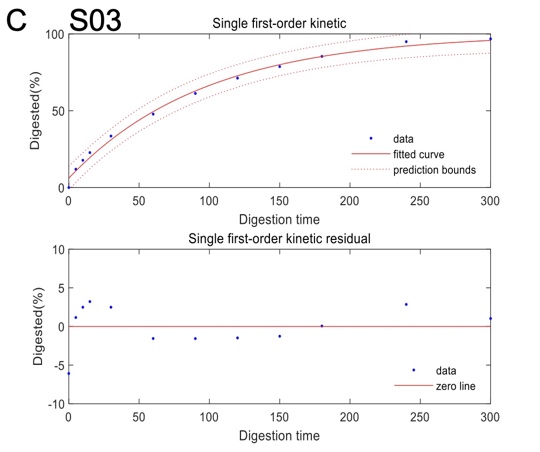

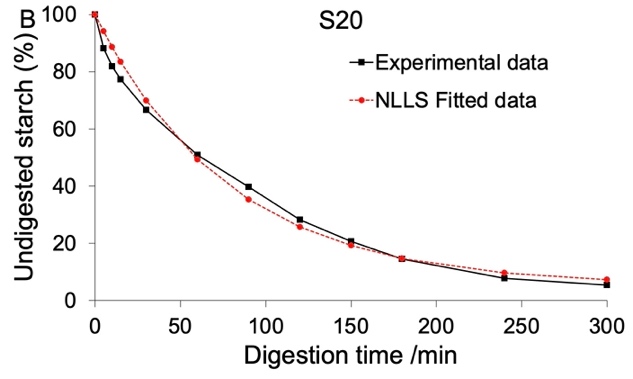

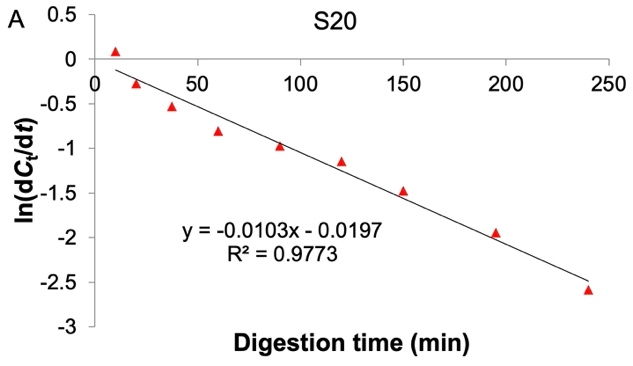

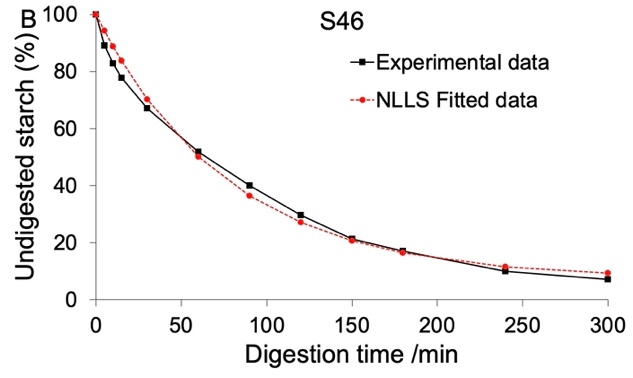

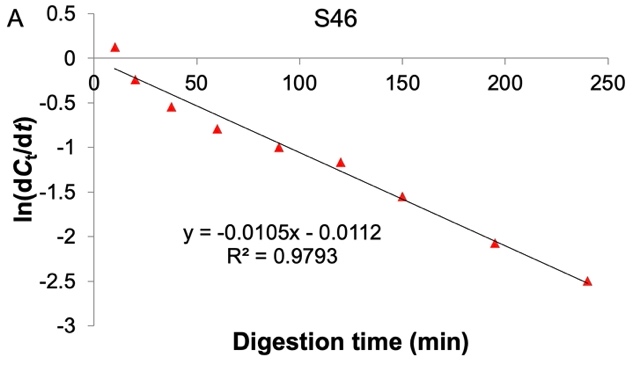

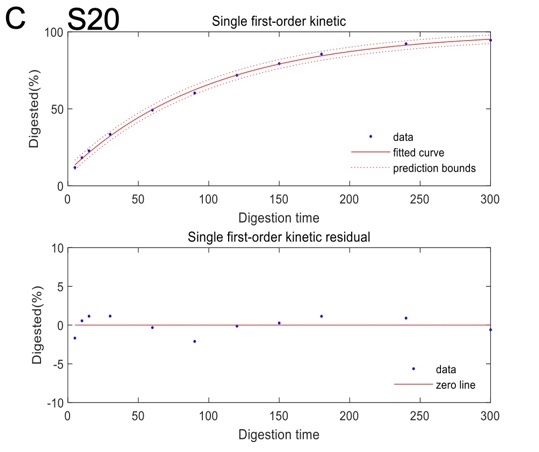

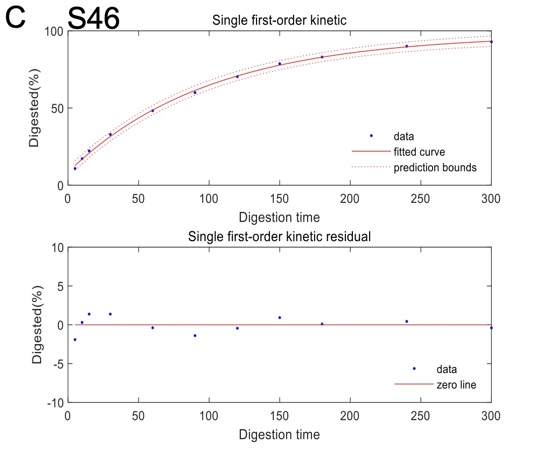


Supplementary Figure 3. Typical fitting of digestion plots to starch digestion models for AWR (S01-S03) and DRs (*indica* rice: S46; *japonica* rice: S20), (A) logarithm of slopes (LoS), (B) non-linear least-squares (NLLS) and (C) parallel model.
